# Young fishes persist despite coral loss on the Great Barrier Reef

**DOI:** 10.1038/s42003-019-0703-0

**Published:** 2019-12-06

**Authors:** Sharon Wismer, Sterling B. Tebbett, Robert P. Streit, David R. Bellwood

**Affiliations:** ARC Centre of Excellence for Coral Reef Studies and College of Science and Engineering, James Cook University, Townsville, QLD 4811 Australia

**Keywords:** Climate-change ecology, Tropical ecology, Ecosystem ecology

## Abstract

Unprecedented global bleaching events have led to extensive loss of corals. This is expected to lead to extensive losses of obligate coral-dependent fishes. Here, we use a novel, spatially-matched census approach to examine the nature of fish-coral dependency across two mass coral bleaching events. Despite a >40% loss of coral cover, and the ecological extinction of functionally important habitat-providing *Acropora* corals, we show that populations of obligate coral-dependent fishes, including *Pomacentrus moluccensis*, persisted and – critically – recruitment was maintained. Fishes used a wide range of alternate reef habitats, including other coral genera and dead coral substrata. Labile habitat associations of ‘obligate’ coral-dependent fishes suggest that recruitment may be sustained on future reefs that lack *Acropora*, following devastating climatic disturbances. This persistence without *Acropora* corals offers grounds for cautious optimism; for coral-dwelling fishes, corals may be a preferred habitat, not an obligate requirement.

## Abstract (web summary)

Sharon Wismer et al. report the effects of coral loss on the Great Barrier Reef on coral-dependent fish populations. They show that despite huge losses in coral cover, both adult and young fishes persisted by using a range of alternative reef habitats.

## Introduction

Coral reefs are one of the world’s best examples of a high diversity system, exhibiting extensive networks of interdependencies, where species often live in close proximity to one another (e.g., refs. ^1,2^). Beyond symbioses, one of the most widespread and visually apparent interactions is the close association between reef fishes and corals. Indeed, many reef fishes are regarded as ‘coral dependent’. This is a particularly common trait among damselfishes, which are typically classified as obligate or facultative live coral dwellers^3^. Distinctions between these categories are based on the frequency of individuals occupying live coral hosts, i.e., >80% and <30%, respectively^3^. A greater reliance on live corals is therefore expected in fishes with more frequent or permanent coral associations^3^. Global coral loss, as a result of climate-induced mass coral bleaching, therefore, raises questions over the fate of these fishes, in particular, fishes with so-called obligate live coral associations.

Today’s post-bleaching environments foreshadow the future of coral reefs, where selective coral mortality results in novel reef types, characterised by an absence of *Acropora* corals and an associated decrease in structural complexity^4–7^. The loss of critical, habitat-forming *Acropora* is expected to have negative impacts for reef fishes, especially obligate coral-dependent fishes, due to the loss of three-dimensional physical structure, food and chemical cues^3,8–10^. Indeed, by definition, the term ‘obligate’ suggests that living corals are ‘biologically essential for the survival’ of these fishes^11^. Thus, these iconic, obligate coral-dependent reef fishes are expected to be extraordinarily vulnerable to the loss of their preferred corals. Recent back-to-back bleaching events on the Great Barrier Reef (GBR)^12^ offer a unique opportunity to explore the implications of coral loss for this iconic relationship.

Lizard Island, in the northern region of the GBR, had experienced extensive declines in the cover of key coral species, due to cyclone damage and crown-of-thorns starfish outbreaks (e.g., refs. ^13,14^). Remaining pockets of high coral cover were predominately situated within the sheltered lagoon and represented a ‘last refuge’ for large coral stands and *Acropora* colonies. Within these last refuges, we documented changes in the cover of live coral and the abundance of reef fishes, across a 24-month (2016–2018) sampling period, which encompassed two unprecedented consecutive mass coral bleaching events and two fish recruitment periods. Specifically, our novel sampling methodology^15^ was designed to provide high-resolution quantification of both fishes and corals, in 132 spatially matched photoquadrats (each 1 m^2^), spread across the ~10 km^2^ reefal system of Lizard Island, Australia.

In response to two consecutive mass coral bleaching events, total live coral cover at Lizard Island decreased by >40% within our 24-month sampling timeline. As expected, heat-sensitive *Acropora* corals were affected disproportionally, decreasing in cover by over 99%. Despite these devastating losses, and the documented dependency of obligate coral-dependent fishes on branching *Acropora* corals^3,9^, adult populations of ‘obligate’ coral-dependent fishes persisted and recruitment was largely sustained. These results suggest that obligate coral-dependent fishes are far more behaviourally flexible than previously assumed, giving hope that these fishes will continue to persist on climate-impacted reefs of the future, despite the reported tight dependency on *Acropora* corals.

## Results

### Local ecological extinction of *Acropora* corals

**Fig. 1:**
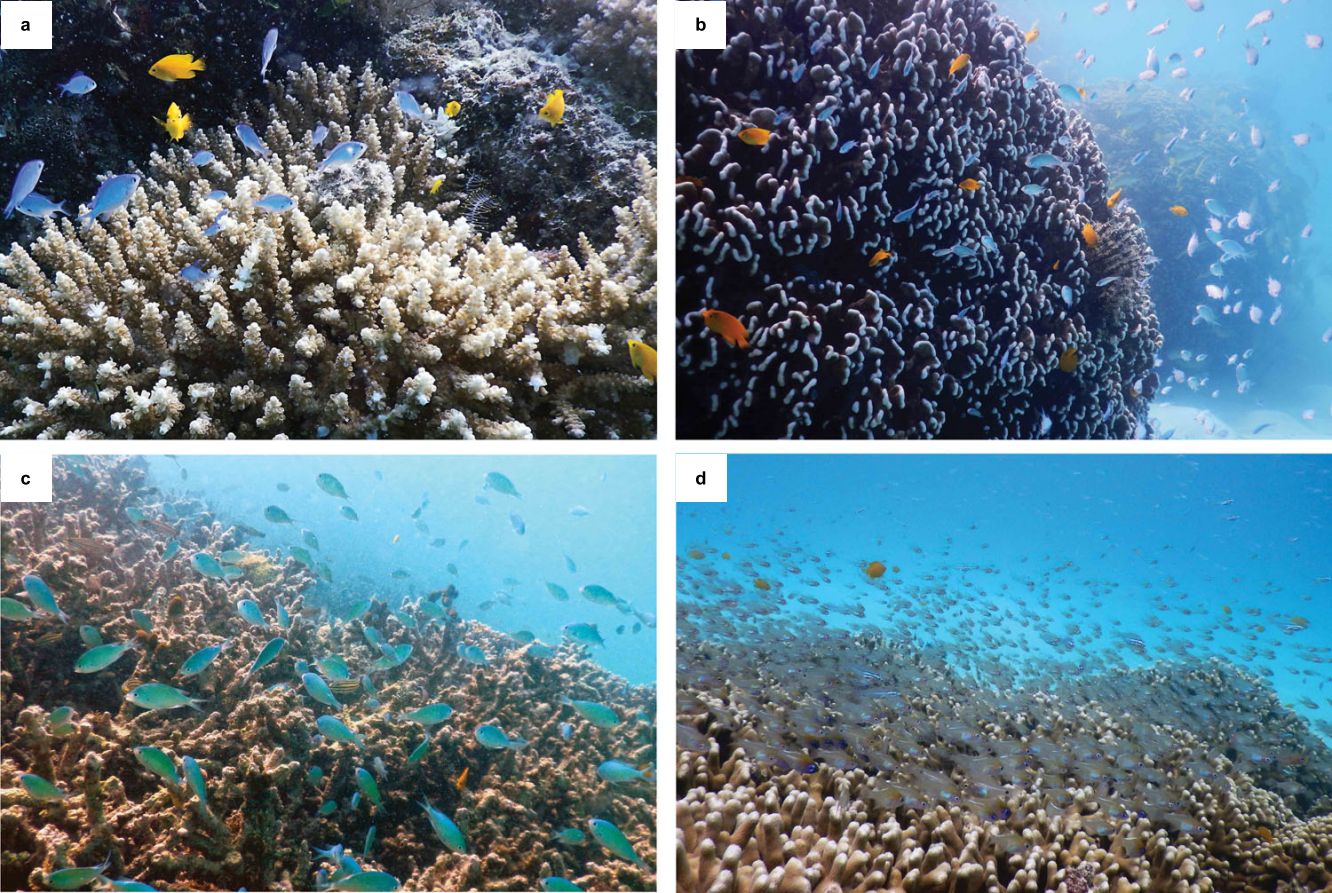
Fish-coral associations. **a** The expected association between fishes and *Acropora* and the ability of fishes to use both **b** alternative coral species (i.e., *Heliopora*) and **c** dead coral substrata. **d** Post-bleaching fish densities remain high (incl. Apogonidae). Photographs: R.P.S. and S.B.T., Lizard Island, January 2018.

**Fig. 2:**
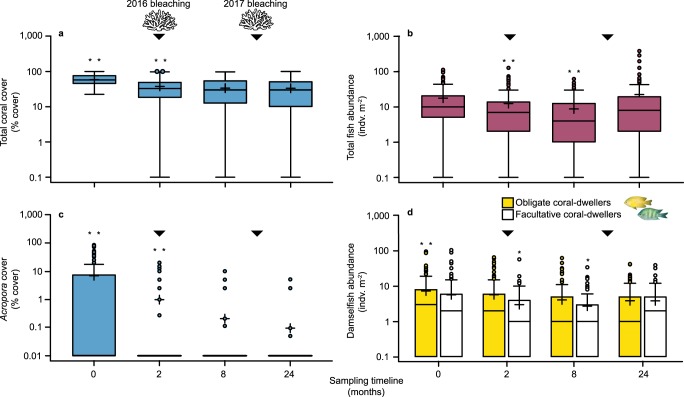
Variation in live coral cover and the abundance of fishes following consecutive mass bleaching at Lizard Island. Changes over 24-months in **a** total coral cover, **b** total fish abundance, **c** *Acropora* cover and **d** damselfish abundance. Boxplots: box limits show the interquartile range; whiskers show 1.5× interquartile range; circles show outliers; and black cross shows the mean. *N* = 132 photoquadrats. Note: log scale on *y*-axes. Arrows: bleaching events (February–April 2016; January–March 2017). Coral cover includes all nominal coral-like taxa (orders: Scleractinia, Helioporacea, Alcyonacea, Corallimorpharia; class: Hydrozoa—*Millepora* spp). Composition of total fish abundance at 24-months is provided in Supplementary Table 4. Damselfishes only include species with an obligate or facultative live coral dependency, following^3^. Asterisks: significant differences in cover/abundance vs. the last sampling period, *P < 0.05, **P < 0.001.

We examined the relationships between corals and associated reef fishes (Fig. 1) over a 24-month sampling period (Supplementary Fig. 1). Our high-resolution photoquadrat methodology (Supplementary Notes 1, Supplementary Table 1, Supplementary Figs. 2 and 3) documented a further 43.1% decrease in total live coral cover at Lizard Island. This decrease was dominated by a loss of *Acropora* cover, which fell by over 99% (Fig. 2a, c). As expected, these heat-sensitive *Acropora* corals showed the strongest, significant collapse, from rare to virtually absent (generalised linear mixed effects models; GLMM; *p* < 0.001; Supplementary Table 2), ranging in cover from 6.9% ± 1.3 (mean ± s.e.) before bleaching to 1.0% ± 0.3 during bleaching and 0.2% ± 0.1 per m^2^ 6-month post-bleaching (Fig. 2c). After the second bleaching event, *Acropora* cover decreased to just 0.1% ± 0.1 cover per m^2^ (Fig. 2c). Other coral genera that are similarly dominated by complex or branching growth forms, such as *Seriatopora* and *Stylophora*, also showed strong declines in cover (Supplementary Fig. 4), while the cover of the branching *Pocillopora* genus remained consistently very low (~1% cover) across all sampling periods (within this genus, the species *Pocillopora damicornis* is a preferred habitat for many obligate coral-dwelling damselfishes; this species remained stable yet exceedingly rare, i.e., < 0.1% cover; Supplementary Note 2). The coral genera *Porites* and *Echinopora* contain a few branching coral species (e.g., *Porites cylindrica*, *Echionopora lamellosa*) that persisted throughout the two bleaching events (Supplementary Fig. 4).

Given the reported tight associations between corals and coral-dwelling reef fishes, and our documented substantial losses of preferred branching coral genera, one would predict a precipitous loss of coral-dependent fishes and the ecological extinction of obligate coral-dwelling fishes, including *Chromis ternatensis*, *Chromis viridis*, *Dascyllus aruanus*, *Dascyllus reticulatus* and *Pomacentrus moluccensis* (Supplementary Table 3, Supplementary Notes 2). Most importantly, one would expect limited recruitment or replenishment of these obligate coral-dwelling species, i.e., species that require live coral habitats for settlement (Supplementary Notes 3).

### Fish responses to coral loss

Remarkably, however, more than half (53.2%) of the obligate coral-dwelling damselfishes persisted. Of the facultative coral-associated damselfishes, 67.6% persisted, while total fish abundance increased by 26.6% 24-month post-bleaching (Fig. 2b, d), the latter primarily due to an increase in cardinalfishes (Apogonidae; see Supplementary Table 4 for fish composition). Although the abundance of coral-associated damselfishes with a facultative or obligate live coral association decreased substantially in response to the first bleaching event (6-months), by 52.2% and 43.9%, respectively (GLMM; *p* < 0.001; Supplementary Table 2; Fig. 2d), we documented no further significant declines following the second bleaching event in 2017 (Fig. 2d; Supplementary Table 2). Indeed, the abundance of facultative and obligate coral-dwelling damselfishes increased by 41.5% and decreased by 5%, respectively, relative to post-2016 (6-months) bleaching levels. Coral-associated fish populations, therefore, appear to have stabilised after 24-months (Fig. 2d; Supplementary Table 2). This suggests that the tight dependency between corals, especially of the genus *Acropora*, and coral-associated fishes may not be as strong as previously assumed.

### Fish recruitment and population persistence

These results show that damselfishes can survive without living corals of their reportedly preferred genus *Acropora*. Critically, however, the future of coral reefs is dependent on fish recruitment and the subsequent survival of juveniles, for the replenishment of local fish populations. Corals, particularly *Acropora*, are a key component of present-day coral reefs^16^, providing fish recruits with important settlement habitats (or settlement cues), as well as a refuge from predators^9,10,17,18^ (Supplementary Notes 3). Indeed, aquaria-based experiments suggest that juvenile fishes (15 species across 6 families), significantly preferred chemical cues from *Acropora* over all other coral genera examined^10^. More broadly, field observations suggest that the loss of live corals, including *Acropora*, will lead to a substantial decrease in fish recruitment, especially in specialised species with strong preferences for, or an obligate association with, live corals (e.g., refs. ^9,17,18^).

**Fig. 3:**
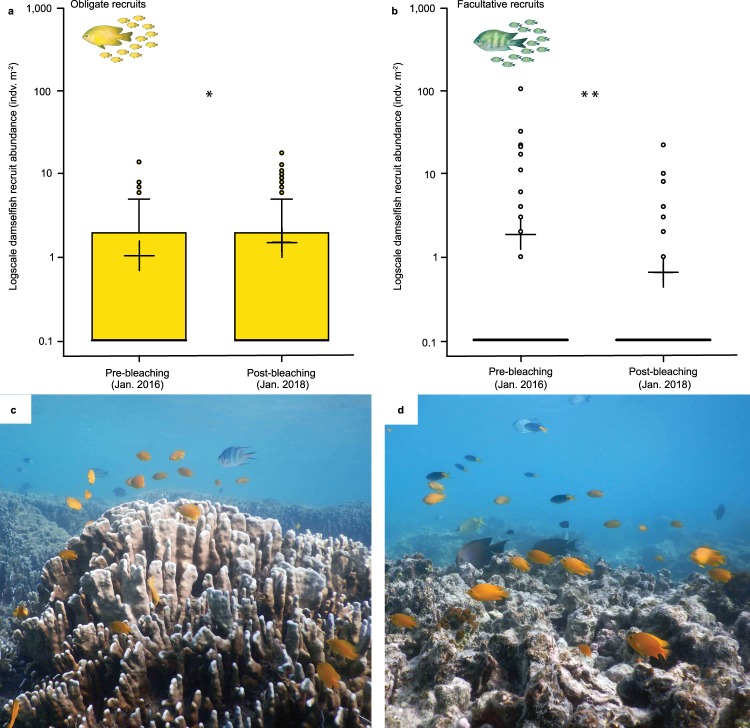
Post-bleaching recruitment of coral-associated damselfishes: changes in recruit abundance and associations with non-preferred microhabitats. Abundance of **a** obligate and **b** facultative coral-dwelling damselfish recruits before and after consecutive mass bleaching events. Boxplots: box limits show the interquartile range; whiskers show 1.5× interquartile range; circles show outliers; and black cross shows the mean. *N* = 132 photoquadrats. Note: log scale on *y*-axes. **c** Coral-associated fishes (adults and recruits) above a less-preferred coral (*Heliopora*) and **d** dead coral substrata. Coral dependencies classified following^3^. Asterisks: significant differences in abundance between sampling periods, *P < 0.05, **P < 0.001. Photographs: S.B.T. from Lizard Island, April 2019.

Remarkably, when we partitioned out recruits, we documented a significant increase in the abundance of obligate coral-dwelling damselfish ‘recruits’ (defined as individuals with juvenile colouration and/or <25% of adult maximum size; Supplementary Table 5). Recruits increased in abundance from 1.0 ± 0.17 to 1.5 ± 0.26 individuals per m^2^ (Fig. 3a) across the 24-months and two mass coral bleaching events. Over the same period, the abundance of facultative recruits, however, decreased significantly from 1.8 ± 0.86 to 0.6 ± 0.20 individuals per m^2^ (Supplementary Table 5; Fig. 3b). Across all 132 quadrats, we documented no clear relationships between obligate and facultative coral-dwelling damselfish recruits and the cover of: all live corals, *Acropora* or other damselfish-‘preferred’ scleractinian corals (which included key coral genera/species typically with branching morphologies). This applied to both before and after the bleaching events (Supplementary Figs. 5 and 6). Furthermore, the few remaining colonies of *Acropora*, and other preferred coral species, were not ‘crowded’ with coral-associated fishes post-bleaching. At a cover of <0.1%, *Acropora* colonies were sparsely distributed, which may have made settlement or relocation challenging. Instead, fish recruits were found in a range of alternate reef microhabitats, including live, non-branching, non-preferred corals, algal-turf-covered dead corals and coral rubble (Fig. 3c, d).

## Discussion

Superficially, our results are consistent with previous findings (e.g., refs. ^5,6,9,19–21^), in that, *Acropora* and other branching corals were most susceptible to bleaching-induced coral mortality and/or fish abundances decreased in response to coral loss. However, the nature of the fish–coral interactions departed markedly from expectations based on the previous observations. Populations of obligate coral-dependent fishes, including recruits, persisted at Lizard Island, despite a long history of coral loss and recent, catastrophic coral bleaching events. We show that the dependency of obligate coral-associated damselfishes on particular coral genera, either as a juvenile or as an adult, may not hold true in a heavily modified reef system devoid of *Acropora*. Instead, fishes with ‘obligate’ live coral associations appear to be behaviourally flexible; key live coral genera appear to be a preference, not an obligate requirement. This highlights limitations and potentially misleading connotations with the terms ‘obligate’ and ‘coral dependent’. Instead, our findings support the term ‘coral-associated’ fishes.

The persistence of obligate coral-dwelling recruits and juveniles is particularly surprising. Obligate coral-dwelling species often show strong preferences for live *Acropora* settlement habitats (e.g., refs. ^10,17,18^), while consecutive mass bleaching is expected to decrease the abundance^20,22,23^, and potentially, the physical condition (e.g., refs. ^24–26^), of adult spawning fish populations. In combination, this is expected to have grave consequences on the recruitment dynamics of these fishes. Sustained recruitment under these conditions was therefore unexpected.

Larval supply is often stochastic, and can vary substantially between sampling years^27,28^, as seen in the pulse of cardinalfishes at 24-month post-bleaching (Fig. 2b, Supplementary Table 4). Thus, the long-term stability of our recorded recruitment patterns will need to be evaluated over coming years. However, given the evidence to date, it appears unlikely that we simply recorded an ephemeral, stochastically large recruitment pulse of damselfishes after the second bleaching event. This is because: (a) the abundance of coral-associated damselfish recruits appeared to be relatively stable across our 24-month study period; and (b) previous evidence suggests that peak recruitment of *P. moluccensis* is typically in February^29^, while our surveys were largely undertaken in January. Given this timing, it is likely that our surveys underestimated the abundance of some obligate damselfish recruits. The fact that recruitment patterns of obligate coral-dwelling fishes in our study were persistent and stable is therefore very promising, especially given the lack of *Acropora* cover, and the expectation of a reduced larval supply.

Although our results provide a degree of cautious optimism for the future of climate-impacted coral reefs, we acknowledge the limitations imposed by the spatial and temporal scales of our study. Our high-resolution quadrats are comparatively small (1 m^2^), and were sampled across a single reef system on the GBR. The majority of previous studies sample fishes and corals at larger spatial scales (Supplementary Table 1, but see Supplementary Table 6). Additional high-resolution studies, across multiple geographic locations, are needed to determine the generality of our findings. Our high-resolution samples, however, provide a novel perspective, in that we are able to focus on small, site-attached coral-associated fishes, examine fish–coral interactions at the scale of an individual fish’s daily activity and, thus, directly assess widely held assumptions concerning the spatial match between fishes and coral loss^15^ (Supplementary Table 1, Supplementary Notes 4).

In terms of temporal scale, our study evaluated changes across a 24-month time period. However, as the global mass bleaching event only occurred in 2016, we are documenting this phenomenon as it is unfolding and must highlight the need for long-term monitoring. We note the possibility of delayed effects, through density-dependent mortality^30^, or further losses, as a result of coral erosion^9,31,32^ and/or decreased physiological condition of fishes over time^8,26^. Living in degraded or non-preferred microhabitats may also reduce the survivorship^33^ and growth rates^24,34^ of coral-associated fishes, with potential long-term fitness consequences. Nevertheless, after decades of coral loss, repeated cyclones, back-to-back bleaching events and the local ecological extinction of *Acropora*, many obligate coral-associated fishes have persisted at Lizard Island 24-month post-bleaching. Whether this pattern will be sustained in the long-term remains unknown. Recent photographs (Fig. 3), however, taken in April 2019 (another 15-months after our last analysed survey), are promising. Populations of obligate coral-dwelling fishes (i.e., *P. moluccensis*) have continued to persist at Lizard Island, offering hope for the long-term survival of coral-dependent fish species on future *Acropora*-free reefs.

Our study provides a new perspective on fish–coral relationships and offers insights into how fishes may cope with novel reef configurations devoid of *Acropora* corals. Contrary to expectations, both populations and recruitment of obligate coral-associated fishes were maintained across 24-months and two mass bleaching events, despite the local functional extinction of *Acropora* corals. Given the strong associations between obligate live coral dwellers and key coral genera, we expected a stronger response in these fishes, especially after 2 years, when the structural integrity of dead corals had started to deteriorate. These results call for a re-evaluation of fish–coral associations and the ecological functions of *Acropora* within reef systems. *Acropora* has been widely documented to be the preferred habitat choice for a plethora of species (e.g., refs. ^3,35^; Supplementary Table 3) and we commonly assume that specialised fishes with tight links to corals require a living coral of a specific species, genus or growth form. However, when *Acropora*, or other preferred coral genera/species, are not available, these fishes readily associated with a diverse range of alternate habitats that included algal-turf-covered dead corals and rubble. From an evolutionary perspective, *Acropora* dominance is a relatively recent phenomenon^16,36^, suggesting that other reef habitats sufficed in the past. This may also hold true for future reef configurations. Although *Acropora* may currently be the preferred choice for many coral-associated fishes, it does not appear to be a non-negotiable, obligate requirement. We may need to reconsider the extent of obligate associations on coral reefs. On rapidly changing Anthropocene reefs, behavioural flexibility, in ‘obligate’ coral-associated fishes, offers some hope for their future persistence.

## Methods

### Study site and bleaching timeline

This study was conducted at Lizard Island, in the northern GBR, Australia (14°40′S, 145°28′E), across a 24-month sampling period, ranging from January 2016 to January 2018 (Supplementary Fig. 1). During this timeframe, the GBR experienced unprecedented back-to-back mass bleaching of scleractinian corals (i.e., February–April 2016, mean sea surface temperatures (SST) of 29.1, 29.1 and 27.8 °C, respectively; and from January–March 2017, mean SST of 28.8, 28.8 and 28.7 °C, respectively), as a result of prolonged elevated sea-surface temperatures^12^. Bleaching in 2016, severely impacted the northern 1000 km of the GBR, particularly the northern region between Port Douglas and the Torres Strait, while the 2017 bleaching event primarily impacted the reef further to the south, between Townsville and Cooktown^12^. We quantified changes in both coral cover and fish assemblages in response to consecutive mass bleaching across four sampling periods: (1) January–February 2016 (i.e., prior to mass bleaching), (2) April 2016 (i.e., during the peak of the first bleaching event), (3) October 2016 (i.e., 6-months after the peak of the first bleaching event) and in (4) January 2018 (i.e., 20-months after the peak of first bleaching event; ~10-months after the second bleaching event).

### Sampling methodology

We employed a novel sampling methodology (Supplementary Notes 1) specifically designed to minimise diver effects, while yielding replicate, high-resolution counts of small, visually apparent coral-associated reef fishes (comprehensive methodological description provided in^15^). This method used a series of ‘photoquadrats’ of the same 1 m^2^ area across the entire 24-month sampling period, in which both coral cover and reef fishes were quantified simultaneously. Hence, each specific 1 m^2^ quadrat area was sampled four times. This method, therefore, provides a direct quantification of small-scale spatial overlap for both corals and fishes over time.

In total, we surveyed 19 locations across Lizard Island, predominately within the lagoon (Supplementary Fig. 3). At each site, divers swam a transect (range: 50–210 m; transect length dependent on individual reef length) along the reef ‘crest’ (at 0–4 m below chart datum; sites were chosen haphazardly) taking photographs of a 1 m^2^ quadrat (Camera: Nikon Coolpix AW130) at ~5 m intervals (range: 12–38 quadrats per indv. transect). Reef ‘crest’ habitats were chosen as they typically boast high coral cover^37^. Each replicate quadrat location consisted of three images: an undisturbed horizontal perspective photograph of the reef and coral-associated reef fishes (at a distance of ~2 m), taken within seconds of reaching the site and prior to the placement of the quadrat (i.e., reducing the so-called diver effect ; Supplementary Notes 1), ensuring all fishes 1.5 m above the substratum were included in the photograph; a second horizontal perspective photograph with the 1 m^2^ quadrat in place, using the identical camera placement as in the first image; and a planar perspective photograph (i.e., bird’s-eye view) of the 1 m^2^ quadrat in place over the substratum. Upmost care was taken when placing quadrats to not damage corals or other benthic organisms. Disturbance to the fish community by self-contained underwater breathing apparatus (SCUBA) divers was minimal and fish activity returned to normal soon after the photographs were taken. For subsequent sampling periods, transect starting points were identified using global positioning system (GPS) coordinates, while precise 1 m^2^ quadrat locations were identified using a second UW camera containing all previous images as a reference. All quadrat locations were photographed across four sampling periods, at 0, 2, 8 and 24-months. The duration of each sampling trip was ~2 weeks. All transects were conducted on SCUBA by two divers between 09:00 and 16:00 h (all photographs were taken by R.P.S. and S.B.T.). This study was observational in nature, no material was removed and all access was covered by permits granted by the GBR Marine Park Authority.

### Image analysis

Per sampling period, a total of 132 (out of 451) 1 m^2^ photoquadrats were analysed. Since we were explicitly examining the response of coral-associated reef fishes to mass bleaching, only quadrats with a minimum live coral cover of 20% in the first sampling period were analysed. ‘Coral’ is used in the broad sense of the term and refers to taxa in the orders: Alcyonacea, Corallimorpharia, Helioporacea, Scleractinia; class: Hydrozoa (*Millepora* spp.)^38^. To quantify reef fishes, all selected photoquadrats were processed in Adobe Illustrator, by drawing an outline of the quadrat on the first photograph of the series (i.e., undisturbed), using the second photograph in the series as a reference. All visible reef fishes within the delineated 1 m^2^ (and ~1.5 m above the quadrat) were recorded to species level and categorised as either adult or recruit. Recruit was defined as the presence of juvenile colouration and/or <25% of adult maximum size, and therefore, this category included both newly settled reef fishes, as well as juvenile fishes a few months in age. Visually similar species *Chromis viridis* and *Chromis atripectoralis* were grouped into one species category *C. viridis* to avoid misidentification. Coral-associated damselfishes (Pomacentridae) were classified into two categories: obligate coral dwellers and facultative coral dwellers, in accordance to their documented live coral dependency, following^3^ (Supplementary Table 3).

Coral cover was quantified using the third photograph in the series (i.e., bird’s-eye view), using the software photoQuad^39^, which generated 40 randomly stratified points over each photoquadrat. For each point, the underlying benthic covering was recorded, i.e., coral to the lowest taxonomic level (generally genus or species), growth form, bleaching status, etc. Only 16 of 21,120 benthic points examined were categorised as unidentifiable, and were therefore excluded from analyses. The effect of random sampling, as well as variation in quadrat placement, was assessed by examining 40 random points from the first sampling period vs. the exact same 40 points from the third sampling period (October 2016, 6-months after peak bleaching; *n* = 15 randomly selected quadrats). The results showed just a 1.4% difference, and hence, our method appears to provide a good indication of benthic changes among temporal samples (analyses published in 15). For consistency, all images were processed by one person (S.B.T.).

### Statistical analyses

We used GLMMs to test for differences in the proportional cover of total live corals, and *Acropora* spp., as well as the abundance of all fishes, coral-associated (facultative + obligate) damselfishes, facultative coral-dwelling damselfishes and obligate coral-dwelling damselfishes among the four sampling periods, i.e., before, during, 6-months after and 24-months after mass bleaching. Proportional coral cover data were examined using a GLMM with a binomial distribution and, where necessary, fitting an observation-level random effect to account for overdispersion. Fish abundance data were examined using a GLMM with a negative binomial distribution, to account for the non-normal and overdispersed nature of the count data. In all models, sampling period (before, during, 6-months after and 24-months after mass bleaching) was fitted as a fixed effect, while quadrat ID, nested within transect ID, were fitted as random effects, to account for the lack of spatial independence and the repeated measures sampling design. Model fits were evaluated using residual plots.

Data focussing only on the abundance of coral-associated damselfish recruits, and their association with coral cover, were also examined using GLMMs. Initially, the abundance of recruits in all coral-associated damselfish species and recruits in species with an obligate or facultative coral dependency were compared between summer sampling periods (January/February 2016 and January 2018), which aligns with summer recruitment pulses^40^. In this case, zero-inflated GLMMs with a negative binomial error distribution were used to account for the non-normal, overdispersed and zero-inflated nature of the data. In the two models, sampling period (January/February 2016 and January 2018) was fitted as a fixed effect, while quadrat ID nested within transect ID, was fitted as random effects to account for the lack of spatial independence and the repeated measures sampling design. Subsequently, relationships among the abundance of all coral-associated damselfish recruits and coral cover were explored separately for the January/February 2016 and January 2018 sampling trips. The relationships between recruits and coral cover were specifically explored separately between trips to assess if the nature of the relationship had changed. However due to this division, a Bonferroni correction was applied to subsequent models (*α* = 0.025). In these models, coral cover was considered as an explanatory variable in two ways: total coral cover and cover of corals preferred by damselfishes. The corals preferred by damselfishes was based on^3^ and included all *Acropora* spp., *Echinopora lamellosa*, *Echinopora mammiformis*, *Porites* *cylindrica*, *Porites nigrescens*, *Pocillopora damicornis*, *Pocillopora verrucosa* and *Seriatopora hystrix*. GLMMs were all based on a negative binomial error distribution to account for the non-normal and overdispersed nature of the data. Zero-inflated models were utilised to account for the large number of zeroes in the data. An extreme outlier was present in the January/February 2016 data and analysis was performed both with and without this data point. Where the outlier influenced model significance, this is noted in the results. Due to the nature of the *Acropora* spp. cover data, no formal analysis was conducted to examine the relationship between *Acropora* cover and recruit density. Furthermore, the relationship between facultative and obligate recruit density, and the cover of corals was visualised graphically, but again, the nature of the data prohibited formal statistical analysis. Statistical modelling was performed in the software R^41^, using the lme4^42^ and glmmTMB^43^ packages.

### Reporting summary

Further information on research design is available in the Nature Research Reporting Summary linked to this article.

## Supplementary information

Supplementary Information

Description of Additional Supplementary Files

Supplementary Data 1

Reporting Summary

## Data Availability

The data that support the findings of this study are available in the Supplementary Data and from the corresponding author upon request.
