# Supplementary material for: Young fishes persist despite coral loss on the Great Barrier Reef
Source: Commun Biol. 2019 Dec 6;2:456. doi: 10.1038/s42003-019-0703-0 (PMC6898333; doi:10.1038/s42003-019-0703-0)
Supplement: Supplementary file 1 — Supplementary Information [file 42003_2019_703_MOESM1_ESM.docx]

Supplementary Information

**Supplementary Figures**

*
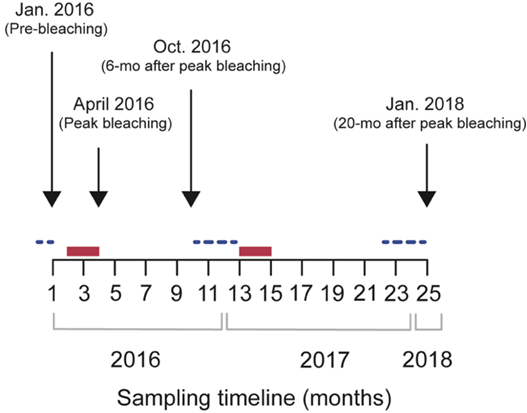
*

**Supplementary figure 1. Sampling timeline.** Arrows: completed sampling trips; Red bars: duration of marine heatwaves that resulted in mass coral bleaching; Dashed blue lines: peak reef fish replenishment (larval supply) to Lizard Island (1); Numbers indicate months since the first sampling period, i.e. 1 = January 2016, etc.).

**
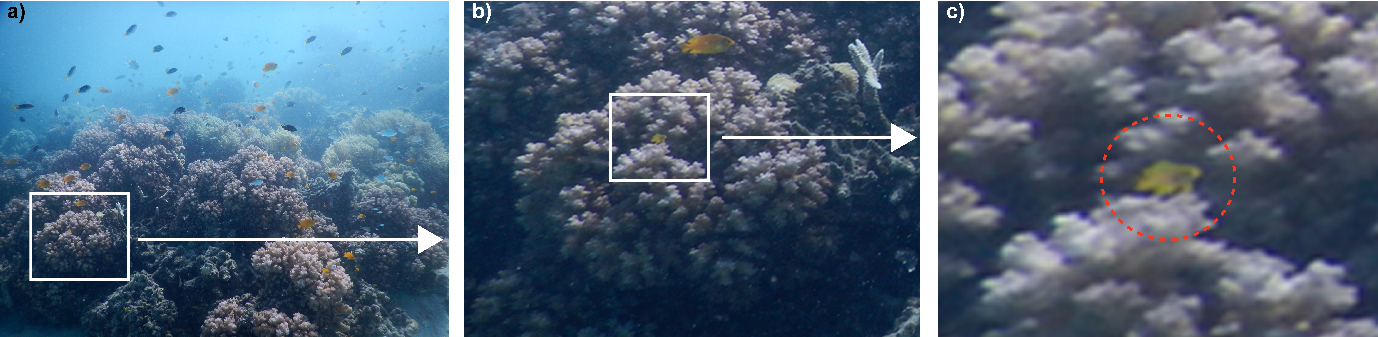
**

**Supplementary figure 2. Examples of images used to quantify changes in reef fish abundance and species richness, in response to consecutive mass bleaching.** Panel images (2a, b, c) show increasing levels of magnification of the same photograph, with close-up views of individual reef fishes, including both adults and individuals in the ‘recruit/juvenile’ category (2c juvenile *Pomacentrus* *moluccensis*). Note, white squares do not represent the quadrat, but areas of increasing magnification (cf. 2).


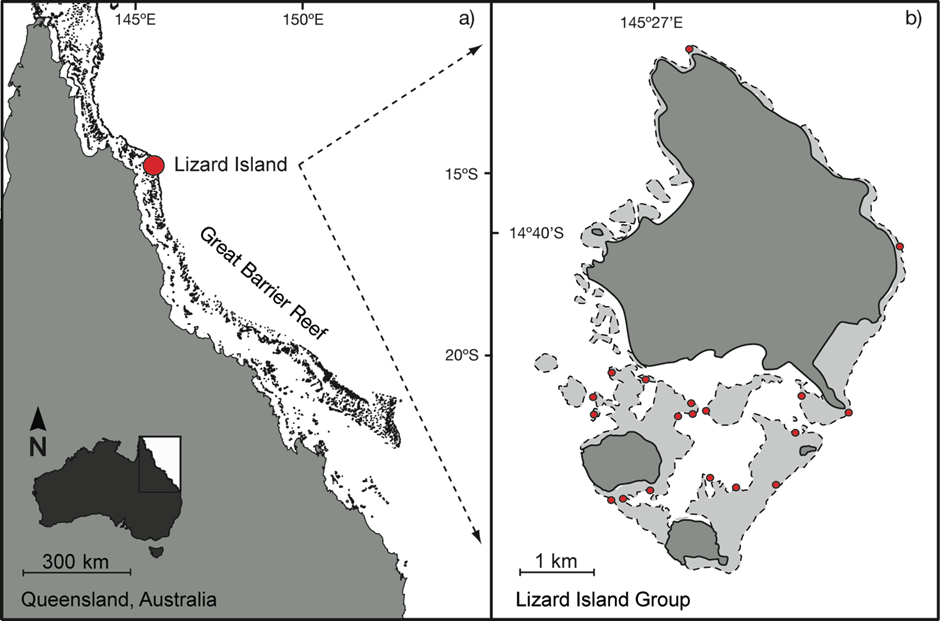


**Supplementary figure 3. Map of study sites.** a) Queensland, Australia (filled grey) and the Great Barrier Reef (GBR). Lizard Island (filled red circle) is located in the remote, northern region of the GBR; b) Lizard Island Group (filled dark grey) and individual study sites (n = 19; filled red circles). Each site indicates the locatia)on of an individual transect (middle of transect). Dashed-light grey areas represent reef habitats. Maps were traced from publically available resources provided by the Great Barrier Reef Marine Park Authority (GBRMPA).

**
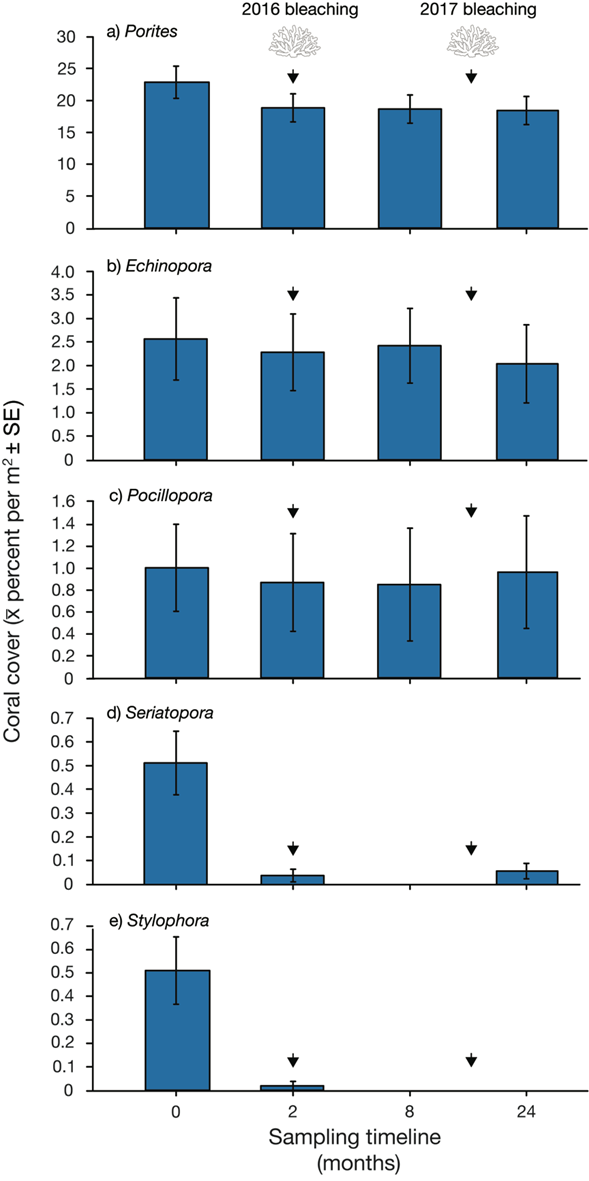
**

**Supplementary figure 4.** **Temporal variation in the cover of common coral genera following consecutive mass bleaching at Lizard Island, Australia.** Changes in a) *Porites*, b) *Echinopora*, c) *Pocillopora*, d) *Seriatopora* and e) *Stylophora*, sampled across a 24-month timeframe. Mass bleaching events (indicated by arrows) occurred between Feb. and April of 2016 and Jan. and March 2017. Values represent means ± Standard Error (SE).

**
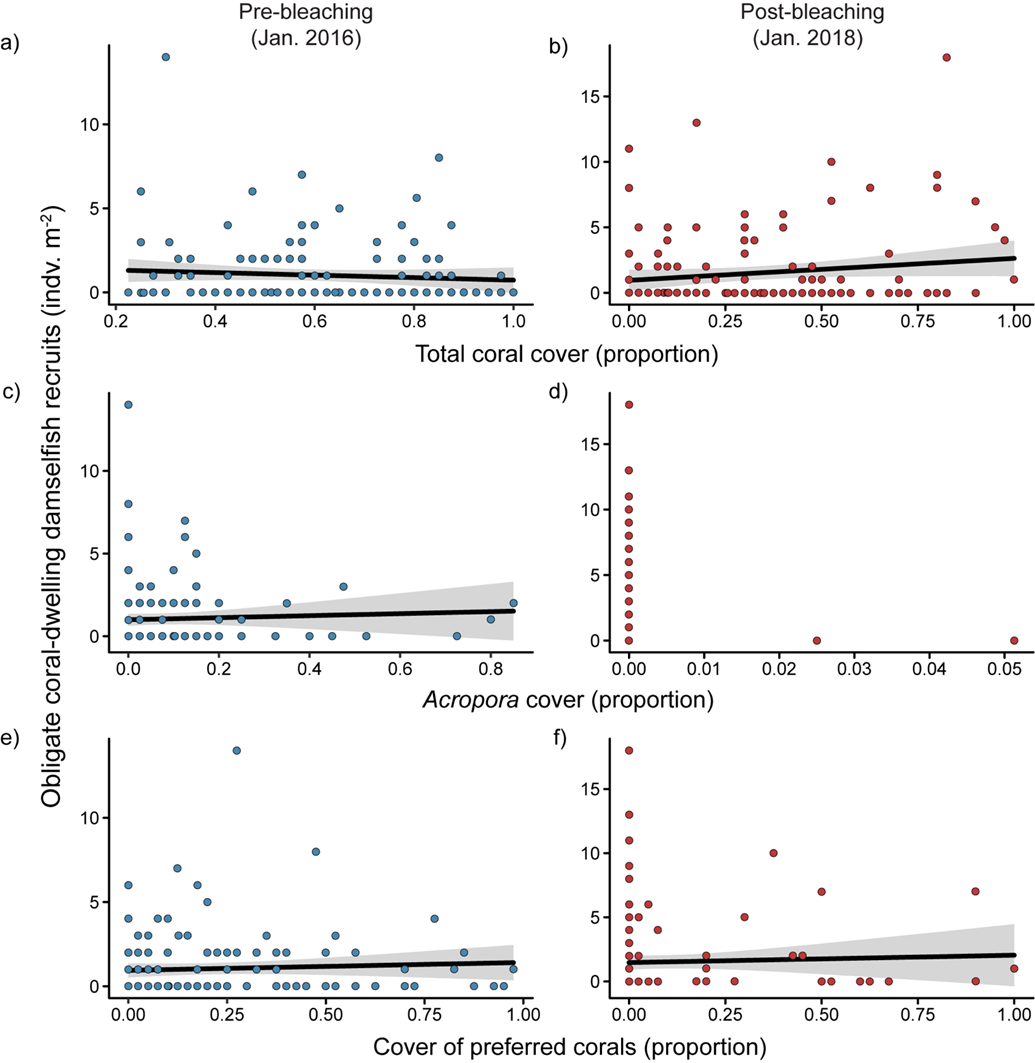
**

**Supplementary figure 5.** Relationships between obligate coral-dwelling damselfish recruits and coral cover: a, b) total coral, c, d), *Acropora* and e, f) preferred corals, before (Jan. 2016) and after (Jan. 2018) consecutive mass bleaching. Shaded areas: 95% confidence intervals.


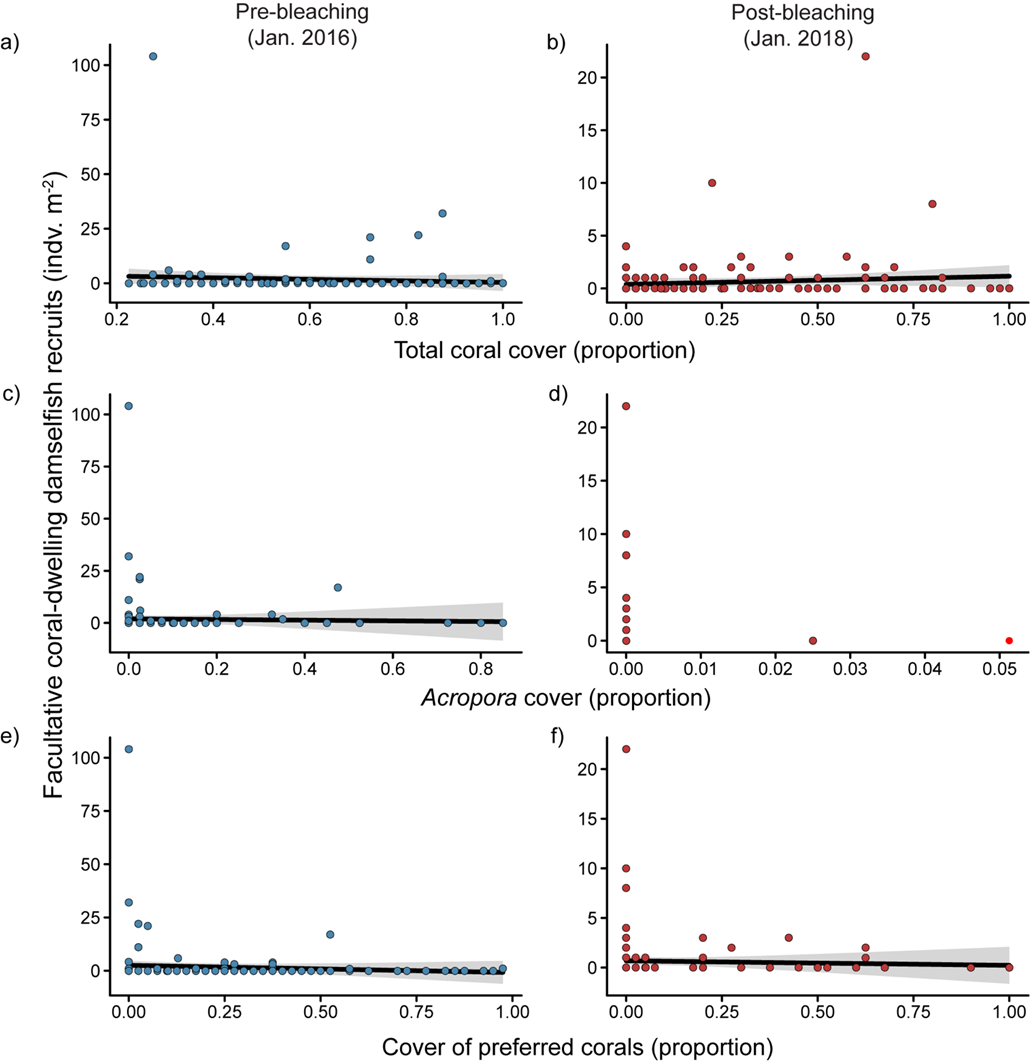


**Supplementary figure 6.** Relationships between facultative coral-dwelling damselfish recruits and coral cover: a, b) total coral, c, d), *Acropora* and e, f) preferred corals, before (Jan. 2016) and after (Jan. 2018) consecutive mass bleaching. Shaded areas: 95% confidence intervals.

**Supplementary Tables**

**Supplementary table 1. Methodological comparison of published studies that assessed the responses of both corals and fishes to mass bleaching.** Time: duration since mass bleaching event. Methods: units used to quantify Fishes (F) and Corals (C); Spa/Temp: a sampling design that provided a direct spatial and temporal overlap, i.e. both fishes and corals were quantified within the same spatial scale (i.e. identical m^2^ area), and these precise areas were repeatedly sampled across time (i.e. before and after mass bleaching). Juv/Rec: study quantified the abundance of juvenile/recruit fishes. WA: Western Australia; rep: replicate; T: transects; *: study evaluated fish recovery across 30 years, using yearly samples from 1980 to 2010.

| **Published study** | **Bleaching** | **Loc.** | **Time** | **Method** | **Spat/Temp** | **Juv/Rec** |
| --- | --- | --- | --- | --- | --- | --- |
| 01. Wellington & Victor (1985)^3^ | 1982/1983 | Panama | 1-yr | F: rep. 30m x 0.5m belt T  C: rep. 30m line-intercept T  only quantified *Stegastes acapulcoensis* (F) | - | Yes |
| 02. Shibuno et al. (1999)^4^ | 1998 | Japan | 2-mo | F: 100m x 4m belt T (using 100m perm. line)  C: rep. 25m line-intercept transects | - | - |
| 03. Lindahl et al. (2001)^5^ | 1997/1998 | Tanzania | 6-mo | F: rep. experimental plots (2.5m^2^)  C: exp. plots (2.5m^2^) of transplanted *Acr. formosa* | Yes | - |
| 04. Kokita & Nakazono (2001)^6^ | 1998 | Japan | 2-mo | F: quantified tagged fish in 30m x 40m quadrat  C: Visual assessment/semi-quantitative  only quantified *Oxymonacanthus longirostris* (F) | Yes | - |
| 05. Adjeroud et al. (2002)^7^ | 1991, 1994 | Moorea | 6-yrs | F: rep. 25m x 4m quadrats  C: rep. 25m point-intercept T (every 0.25m) | - | Yes |
| 06. Booth & Beretta (2002)^8^ | 1998 | GBR | 1-yr | F: rep. 15m x 1.5 to 4m belt T  C: rep. 15m video T, pausing every 50 frames, ea 0.4m^2^  only quantified damselfishes (F) | - | Yes |
| 07. McClanahan et al. (2002)^9^ | 1998 | Kenya | 3-yrs | F: rep. 500m^2^ belt T  C: rep. 10m line-intercept T | - | - |
| 08. Riegl (2002)^10^ | 1996, 1998 | Dubai | 2-yrs | F: rep. point counts in 7.5m radius cylinder  C: rep. T: 10m line-intercept, 50m point-intercept | - | - |
| 09. Spalding & Jarvis (2002)^11^ | 1998 | Seychelles | 1-yr | F: rep. 20min point counts in an area of 10m diameter  C: Semi-quantitative simple visual assessment of cover | Yes | - |
| 10. Sheppard et al. (2002)^12^ | 1998 | Chagos | 3-yrs | F: rep. 20min point counts in 10m diameter area  C: rep. line-intercept T (length not provided) | - | - |
| 11. Munday (2004)^13^ | 1998 | PNG | 5-yrs | F: rep. 10m x 1m T  C: rep. 10m x 1m T (all colonies of *Acropora* only)  only quantified *Gobiodon* (F) | - | - |
| 12. Pratchett et al. (2004)^14^ | 2002 | GBR | 4-mo | F: rep. 50m x 4m belt T  C: rep. 10m line-intercept T  only quantified *Chaetodon lunulatus* (F) | - | - |
| 13. Sano (2004)^15^ | 1998 | Japan | 1-yr | F: rep. 20m x 1m belt T  C: Qualitative assessment | - | - |
| 14. Garpe et al (2006)^16^ | 1998 | Tanzania | 6-yrs | F: rep. experimental plots (2.5m^2^)  C: same experimental plots (2.5m^2^)  only transplanted *Acr. formosa* (C) | Yes | - |
| 15. Jones et al. (2004)^17^ | 1997/1998  2000/2001 | PNG | 6-yrs | F: rep. 50m x 1 to 4m belt T  C: rep. 50m line T (using 100 random points per transect) | - | Yes |
| 16. Pratchett et al. (2006)^18^ | 2002 | GBR | 3-yrs | F: rep. 50m x 4m belt T  C: rep. 10m line-intercept T  only quantified *Chaetodon* spp (F) | - | - |
| 17. Graham et al. (2007)^19^ | 1998 | Seychelles | 7-yrs | F: rep. 7m radius point counts  C: visual estimation of count area | - | - |
| 18. Wilson et al. (2008)^20^ | 2000 | Fiji | 6-yrs | F: rep. 7 m radius point counts  C: rep. 500cm^2^ digital photos; rep. 30m point intercept T | - | - |
| 19. Graham et al. (2009)^21^ | 1998 | Seychelles | 7-yrs | F: 16x 7m radius point counts  C: replicate 10m line-intercept transects  only quantified butterflyfishes | - | - |
| 20. Gilmour et al. (2013)^22^ | 1998 | WA | 6-yrs | F: rep. 250m permanent T  C: photographs from adjacent 250m T + other 120m sites | - | - |
| 21. Brooker et al. (2014)^23^ | 2011 | GBR | 1-yr | F: rep. 50 x 1m belt T  C: rep. 10m line intercept T  only quantified *Oxymonacanthus longirostris* (F) | - | - |
| 22. Glynn et al. (2014)^24^ | 1982/83  1997/98 | Panama | 30-yrs* | F: rep. permanent 20 x 40m T  C: rep. fixed chain T & 1m^2^ plots, single 4m x 5m^2^ plot | - | - |
| 23. Mangubhai et al. (2014)^25^ | 2002 | C. Pacific | 3-yrs | F: rep. point counts in 7m radius  C: rep. 4m^2^ photquadrats | - | - |
| 24. Richardson et al. (2018)^26^ | 2016 | GBR | 6-mo | F: rep. 30m T (either 1m wide or 5m wide)  C: rep. 30m point intercept T | - | - |
| 25. Stuart-Smith et al. (2018)^27^ | 2016 | GBR | 1-yr | F: rep. 50m x 5m T ‘blocks’  C: rep. photoquadrats every 2.5m along T | - | - |
| 26. Keith et al. (2018)^28^ | 2016 | Indo-Pac. | 1-yr | F: rep. 50x x 5m belt transects  C: rep. 50m point-intercept transects (every 0.5m)  Only quantified *Chaetodon* species | - | - |
| 27. Wismer et al. (2019)^2^ | 2016 | GBR | 6-mo | F: rep. 1m^2^ quadrats  C: rep. 1m^2^ quadrats (same plots as above) | Yes | - |
| 28. Wilson et al. (2019)^29^ | 1998/2016 | Seychelles | 7-yrs/1-yr | F: 154 m^2^ replicate areas (7 m radius)  C: replicate 10m line-intercept transects | - | - |
| 29. McClure et al. (2019)^30^ | 2016 | GBR | 6-mo | F: rep. 10m timed swims with 5m belt; 50m x 5 m belt T  C: rep. 10m and 50m point and line-intercept T | - | - |

**Supplementary table 2**. Summary of generalised linear mixed effects model (GLMM) results used to examine difference in the abundance of reef fishes and coral cover across four sampling periods encompassing both the 2016 and 2017 mass coral bleaching events at Lizard Island, Australia. SE = standard error, df = residual degrees of freedom, 2.5% and 97.5% = confidence intervals. The sampling period two years post 2016 bleaching is fitted as the reference level. Sampling month 0 = before; month 2 = during; month 8 = after.

| Response variable | Model | Predictor variable | df | Effect size | 2.5% | 97.5% | SE | *z* value | *P* |
| --- | --- | --- | --- | --- | --- | --- | --- | --- | --- |
|  |  |  |  |  |  |  |  |  |  |
| Total coral cover | Binomial (GLMM) | Intercept  Before  During  After | 521 | -0.9740  1.4586  0.3295  0.0276 | -1.3283  1.2642  0.1340  -0.1694 | -0.6197  1.6529  0.5250  0.2246 | 0.1808  0.0992  0.0997  0.1005 | -5.388  14.708  3.304  0.274 | **< 0.001**  **< 0.001**  **< 0.001**  0.7839 |
|  |  |  |  |  |  |  |  |  |  |
| *Acropora* coral cover | Binomial  (GLMM) | Intercept  Before  During  After | 522 | -9.7360  4.8802  2.4379  0.8043 | -11.0351  3.9833  1.5117  -0.2601 | -8.4369  5.7772  3.3642  1.8687 | 0.6628  0.4576  0.4726  0.5431 | -14.689  10.664  5.159  1.481 | **< 0.001**  **< 0.001**  **< 0.001**  0.139 |
|  |  |  |  |  |  |  |  |  |  |
| Total no. of reef fishes (individuals) | Negative binomial (GLMM) | Intercept  Before  During  After | 521 | 2.3057  0.0762  -0.4398  -0.7448 | 1.9261  -0.1714  -0.6876  -0.9954 | 2.6853  0.3239  -0.1919  -0.4941 | 0.1937  0.1263  0.1265  0.1279 | 11.904  0.603  -3.478  -5.824 | **< 0.001**  0.5463  **< 0.001**  **< 0.001** |
|  |  |  |  |  |  |  |  |  |  |
| Total no. of coral-associated damselfishes  (individuals) | Negative binomial (GLMM) | Intercept  Before  During  After | 521 | 1.4818  0.4202  -0.0586  -0.3077 | 1.0661  0.1731  -0.3032  -0.5578 | 1.8976  0.6673  0.1859  -0.0577 | 0.2121  0.1261  0.1248  0.1276 | 6.986  3.333  -0.470  -2.412 | **< 0.001**  **< 0.001**  0.6385  **< 0.05** |
|  |  |  |  |  |  |  |  |  |  |
| Total no. of facultative damselfishes  (individuals) | Negative binomial (GLMM) | Intercept  Before  During  After | 521 | 0.9358  0.2342  -0.3853  -0.4493 | 0.5436  -0.0936  -0.7122  -0.7788 | 1.3640  0.5619  -0.0585  -0.1198 | 0.2093  0.1672  0.1668  0.1681 | 4.558  1.400  -2.311  -2.673 | **< 0.001**  0.1615  **< 0.05**  **< 0.01** |
|  |  |  |  |  |  |  |  |  |  |
| Total no. of obligate damselfishes  (individuals) | Negative binomial (GLMM) | Intercept  Before  During  After | 521 | 0.1506  0.5585  0.2599  -0.1463 | -0.4785  0.2643  -0.0340  -0.4499 | 0.7798  0.8527  0.5538  0.1572 | 0.3210  0.1501  0.1500  0.1549 | 0.469  3.721  1.733  -0.945 | 0.6389  **< 0.001**  0.083  0.3448 |
|  |  |  |  |  |  |  |  |  |  |

**Supplementary table 3.** Coral-associated damselfishes recorded in our study, classified according to their live coral dependency and observed coral associations across the Pacific. Obligate dependency: >80% of observed individuals shelter in live coral hosts (31). Facultative dependency: <30% of observed individuals shelter in live coral hosts; fishes that use live coral hosts in an opportunistic or occasional manner and exhibit low fidelity for specific coral hosts (31). *Dischistodus perspicillatus* was grouped as facultative, as it is unknown whether live coral is an obligate or facultative microhabitat requirement for this species (31). P: Planktivore, AF: Algal Farmer, FC: Facultative Corallivore. Specialization, O: Obligate; F: Facultative. Associations are based on coral species reported in 32-34.

| **Fish species** | **Specialization** | **Coral associations** |
| --- | --- | --- |
| *Chromis ternatensis* (P) | O | *Acropora aculeus, A. hyacinthus, A.*  *intermedia, A. jacquelineae, A.*  *kimbeensis, A. longycyanthus, A.*  *muricata, A. paniculata, A. plumosa, A.*  *secale, A. subglabra, A. valenciennesi,*  *A. valida, Isopora palifera* |
| *Chromis viridis* (P) | O | *Acropora cerealis, A. divaricata, A.*  *formosa, A. humilis, A. millepora, A.*  *nasuta, A. pulchra, A. spathulata, A. valida, A. valenciennesi, Echinopora mammiformis, E. lamellosa, Pocillopora damicornis, P. eydouxi, P. meandrina, Porities cylindrica, P. rus* |
| *Dascyllus aruanus* (P) | O | *Acropora aculeus, A. aspera, A. cerealis, A.*  *corymbosa, A. cuneata, A. divaricata, A.*  *formosa, A. humilis, A. longicyathus, A.*  *loripes, A. millepora, A. nasuta, A. pulchra, A. spathulata, A. surculosa, A. tizardi, Echinopora lamellose, E. mammiformis, Heliopora* spp*, Millipora tenella, Montipora digitata, Pocillopora damicornis, P. eydouxi, P. meandrina, P. verrucosa, Porites cylindrica, P. lobata, Seriatopora caliendrum, S. hystrix, Stylophora pistillata* |
| *Dascyllus reticulatus* (P) | O | *Acropora divaricata, A. loripes, A. millepora, A.nasuta, A. secale, A. spathulata, A. valenciennesi,*  *Echinopora mammiformis, E. lamellosa, Pocillopora damicornis, P. eydouxi, Seristopora hystrix, Stylophora pistillata* |
| *Pomacentrus moluccensis* (P) | O | *Acropora aspera, A. caroliniana, A. cerealis, A. digitifera, A. divaricata, A. formosa, A. gemmifera, A. hyacinthus, A. humilis, A. intermedia, A. longycyanthus, A. loripes, A. microclados, A. millepora, A. nasuta, A. palifera, A. paniculata, A. secale, A.selago, A. solitaryensis, A. spathulata, A. tenuis, A. valida, A. valenciennesi, A. yongei, Echinopora lamellosa, E. mammiformis, Heliopora coerulea, Pocillopora damicornis, P. eydouxi, P. meandrina, Porities cylindrica, P. rus, Seriatopora hystrix, Stylophora pistillata* |
| *Abudefduf bengalensis* (P) | F | *Acropora formosa* |
| *Abudefduf sexfasciatus* (P) | F | *Acropora formosa, A. pulchra, Porities cylindrica* |
| *Acanthochromis polyacanthus* (P) | F | *Acropora formosa, A. hyacinthus, Echinopora mammiformis, Porities cylindrica* |
| *Amblyglyphidodon curacao* (P) | F | *Acropora aculeus, A. florida, A. formosa, A. hyacinthus, A. intermedia, A. jacquelineae, A. muricata, A. nasuta, A. paniculata, A. plumosa, A. robusta, A. secale, A. selago, A. solitaryensis, A. valenciennesi, A. valida, Echinopora mammiformis, Isopora brueggemani, I.palifera, Porities cylindrica* |
| *Amblyglyphidodon leucogaster* (P) | F | *Acropora aculeus, A. hyacinthus, A. intermedia, A. jacquelineae, A. muricata, A. paniculata, A. plumosa, A. secale, A. subglabra, A. valenciennesi, Isopora brueggemani* |
| *Chromis margaritifer* (P) | F | Tabular *Acropora* spp |
| *Chrysiptera flavipinnis* (P) | F | *Acropora cerealis* |
| *Dischistodus perspicillatus* (AF) | F | *Pocillopora damicornis, Porites cylindrica* |
| *Hemiglyphidodon plagiometopon* (AF) | F | *Porites cylindrica* |
| *Neoglyphidodon melas* (FC) | F | *Acropora formosa, A. humilis, A. millepora, A. nasuta, A. spathulata, A. tenuis, A. valida, Pocillopora damicornis* |
| *Neopomacentrus azysron* (P) | F | *Acropora caroliniana, A. hyacinthus, A. kimbeensis, A.muricata, A. nasuta, A. paniculata, A. plumosa, A. selago, A. solitaryensis, A.valenciennesi, A. valida, Isopora* spp*, Porites cylindrica* |
| *Neopomacentrus cyanomos* (P) | F | *Pocillopora damicornis, Porites cylindrica* |
| *Pomacentrus amboinensis* (P) | F | *Acropora cerealis, A. divaricata, A. millepora, A. nasuta, A. valenciennesi, Echinopora lamellosa, E. mammiformis, Pocillopora damicornis, Portites cylindrica, Seristopora hystrix, Stylophora pistillata* |
| *Pomacentrus bankanensis* (AF) | F | *Acropora formosa, Porities cylindrica* |
| *Pomacentrus chrysurus* (AF) | F | *Acropora* spp |
| *Pomacentrus grammorhynchus* (AF) | F | Branching coral species |
| *Pomacentrus lepidogenys* (P) | F | *Acropora gemmifera, A. tenuis, Pocillopora damicornis, Stylophora pistillata* |
| *Pomacentrus nagasakiensis* (P) | F | *Pocillopora damicornis, Porities cylindrica* |
| *Pomacentrus pavo* (P) | F | *Acropora pulchra, Porites australiensis, P. cylindrica, P. lobata* |
| *Stegastes apicalis* (AF) | F | *Acropora hyacinthus, Porities cylindrica* |
| *Stegastes fasciolatus* (AF) | F | *Acropora pulchra* |
| *Stegastes nigricans* (AF) | F | *Acropora pulchra, A.* spp., *Porites rus, P. cylindrica* |

**Supplementary table 4. Composition of total fish abundance at 24-months.** In the long-term, total fish abundance increased from 17.7 ± 1.9 to a mean abundance of 22.3 ± 4.3 individuals per m^2^, despite back-to-back bleaching events. This result is contrary to expectation and influenced by a stochastic recruitment pulse of apogonids. Fish families and life stages with the five highest abundances in the last sampling period (Jan. 2018) are provided below. Rec. = Recruit; Juv. = Juvenile.

| **Fish family** | **Life stage** | **Mean indv.**  **± SE m^-2^** | **Range** | **Percentage**  **of total**  **(Jan. 2018)** | **Percentage increase/decrease**  **(Jan. 2016 to**  **Jan. 2018)** |
| --- | --- | --- | --- | --- | --- |
| Apogonidae | Rec./Juv. | 8.7 ± 3.3 | 0 - 325 | 39.1 | 8600 |
| Pomacentridae | Adult | 5.7 ± 0.6 | 0 - 35 | 25.5 | -44.7 |
| Apogonidae | Adult | 3.9 ± 1.2 | 0 - 97 | 17.7 | 178.6 |
| Pomacentridae | Rec./Juv. | 2.2 ± 0.3 | 0 - 22 | 10.0 | -24.1 |
| Caesionidae | Adult | 0.7 ± 0.3 | 0 - 33 | 3.3 | 75.0 |

**Supplementary table 5**. Summary of generalised linear mixed effects model (GLMM) results used to examine difference in the abundance of coral-associated recruit damselfishes, as well as their association with different metrics of coral cover. Models were based on data collected before (Jan./Feb. 2016) the 2016 mass coral bleaching event and approximately two years after the bleaching event (Jan. 2018) at Lizard Island, Australia. SE = standard error, df = residual degrees of freedom, 2.5% and 97.5% = confidence intervals. C.A. = coral-associated. (α = 0.025).

| Response variable | Model | Predictor variable | df | Effect Size | 2.5% | 97.5% | SE | *z* value | *P* |
| --- | --- | --- | --- | --- | --- | --- | --- | --- | --- |
|  |  |  |  |  |  |  |  |  |  |
| Total no. of C.D. damsel recruits | Zero-inflated negative binomial (GLMM) | Intercept  Before | 255 | 1.0138  -0.0195 | 0.3657  -0.5232 | 1.6619  0.4841 | 0.3307  0.2570 | 3.066  -0.076 | **< 0.001**  0.9394 |
|  |  |  |  |  |  |  |  |  |  |
| Total no. of facultative coral-dwelling damsel recruits | Zero-inflated negative binomial (GLMM) | Intercept  Before | 255 | -1.0334  2.1250 | -1.8863  -0.8946 | -0.1805  3.3554 | 0.4352  0.6278 | -2.375  3.385 | **< 0.01**  **< 0.001** |
|  |  |  |  |  |  |  |  |  |  |
| Total no. of obligate coral-dwelling damsel recruits | Zero-inflated negative binomial  (GLMM) | Intercept  Before | 255 | 1.1063  -0.7427 | 0.6240  -1.2489 | 1.5886  -0.2365 | 0.2461  0.2583 | 4.496  -2.875 | **< 0.001**  **< 0.01** |
|  |  |  |  |  |  |  |  |  |  |
| C.A. recruit damsels before | Zero-inflated negative binomial (GLMM) | Intercept  Total Coral | 125 | 1.8723  -1.6219 | 0.6736  -3.3204 | 3.0710  0.0765 | 0.6116  0.8666 | 3.061  -1.872 | **< 0.01**  0.0612 |
|  |  |  |  |  |  |  |  |  |  |
| C.A. recruit damsels before | Zero-inflated negative binomial  (GLMM) | Intercept  Dam. Pref. Coral | 125 | 1.2664  -1.2691 | 0.5265  -2.4499 | 2.0063  -0.0884 | 0.3775  0.6024 | 3.355  -2.107 | **< 0.001**  0.0351 |
|  |  |  |  |  |  |  |  |  |  |
| C.A. recruit damsels after | Zero-inflated negative binomial (GLMM) | Intercept  Total Coral | 125 | 1.0039  0.8621 | 0.4993  -0.0283 | 1.5085  1.7525 | 0.2574  0.4543 | 3.900  1.898 | **< 0.001**  0.0577 |
|  |  |  |  |  |  |  |  |  |  |
| C.A. recruit damsels after | Zero-inflated negative binomial (GLMM) | Intercept  Dam. Pref. Coral | 125 | 1.0272  -0.4725 | 0.3945  -1.8759 | 1.6600  0.9308 | 0.3228  0.7160 | 3.182  -0.660 | **< 0.01**  0.5093 |
|  |  |  |  |  |  |  |  |  |  |

**Supplementary table 6. Published studies evaluating the impact of coral mortality on butterflyfishes and/or cryptobenthic species.**

| **Published study** | **Summary** |
| --- | --- |
| Pratchett et al. (2004)^14^ | This study examined the effects of mass bleaching (GBR) on the abundance, diet and physiological condition of the butterflyfish *Chaetodon lunulatus* across a 22-mo timeframe, and specifically, 4-mo post-bleaching. Despite significant coral decline, these fishes used alternate coral species for food and abundances were relatively unaffected. Fishes did, however, show reduced physiological condition post-bleaching. |
| Pratchett et al. (2006)^18^ | This study is a follow-on from Pratchett et al. 2004 and investigated the impacts of mass bleaching (GBR) on the abundance of butterflyfishes 3-yrs post-beaching. Obligate hard coral feeding fishes showed significant declines in the long-term, while fishes with a lesser dependency on live coral showed no significant declines in abundance. |
| Munday (2004)^13^ | This study investigated the impacts of coral loss (PNG), as a result of increased sedimentation and coral bleaching, between 1996/97 and 2003, on *Gobiodon* species. Coral loss led to significant declines of coral-dwelling gobies. Habitat specialists showed greater proportional declines compared to habitat generalists. |
| Berumen & Pratchett (2006)^35^ | This study examined long-term changes of corals and butterflyfishes across a 24-yr timeframe (1979-2003). A range of disturbances occurred during the sampling timeframe, incl. a significant CoTS outbreak in 1980/81. While both corals and butterflyfishes recovered by 2003 (abundances), community composition was significantly altered (i.e. lack of *Acropora*). Recovery was likely prevented due to ongoing disturbances that included coral bleaching and storm damage. |
| Graham et al. (2009)^21^ | This study investigated the effects of coral loss vs. the loss of structural complexity on obligate and facultative coral feeding butterflyfishes (Seychelles). Obligate coral feeding fishes declined significantly in response to the loss of live coral (but no further losses in response to decreases in complexity), whereas, facultative coral feeding butterflyfishes showed no declines in response to live coral loss, but declined in response to the erosion of the reef framework. |
| Bellwood et al. (2006)^36^ | This study evaluated the cryptobethic fish community (incl. gobies) across a 12-yr time frame incorporating the 1998 mass bleaching event (GBR) (6-yrs post-bleaching). Although no changes in abundance, diversity or species richness were detected, there were marked shifts in fish community composition, in response to mass bleaching. |
| Bellwood et al. (2012)^37^ | This study is a follow-on study from Bellwood et al. (2006) and investigated the long-term impacts (13-yrs post-bleaching) of the 1998 mass bleaching event on cryptobenthic fishes. Despite 13-yrs after the 1998 mass-bleaching event, 96 fish generations and increased abundances, cryptobenthic fish communities have not returned to pre-bleaching configurations. |
| Keith et al. (2018)^28^ | This study examined the effects of the 2016 mass bleaching event (central Indo-Pacific) on the behaviour (aggression, feeding) of *Chaetodon* butterflfishes 12-mo post bleaching. Fishes showed no significant decrease in abundance; however, both aggression and the percentage of bites taken from preferred *Acropora* corals decreased significantly. |

**Supplementary Notes, Discussion, Methods**

**Supplementary notes 1. Novel sampling design**

*Methodological advantages*

1) **Spatial-temporal overlap** - Our study employs a novel sampling technique specifically designed to quantify coral cover and provide high-resolution counts of visually apparent reef fishes that exhibit tight associations with living coral. In particular, our study provides a direct spatial-temporal overlap of both fishes and corals, in that, both fishes and corals were quantified in the same exact area (i.e. replicate 1 m^2^ quadrats) and precise quadrat locations were reassessed multiple times across the 24-mo sampling period. This approach allows us to track individual coral colonies and small areas of reef, along with resident fishes, across two unprecedented, back-to-back mass bleaching events on the GBR. This approach is rare in the literature, with the exception of our previously published short-term results (i.e. 2; Supplementary Table S1). The few studies that do provide a direct spatial-temporal overlap of both fishes and corals in response to mass bleaching either utilised experimental plots consisting of transplanted corals of a single species (i.e. did not survey natural communities), only quantified a single fish species, or applied a non-quantitative approach when assessing coral cover (Supplementary Table S1).

2) **Recruitment** - In addition to adult fishes, we also examined the response of newly settled recruits and small juvenile fishes across the same 24-mo sampling period. We quantified all visible recruits/juveniles, regardless of species. This is likewise rare among published studies, which in contrast typically only quantify juveniles of a single species or family (Supplementary Table S1; but see 7,17). To the best of our knowledge, our study provides the first small-scale, full-assembly assessment of both adult fishes and small recruits/juveniles of coral-associated fishes in response to multiple mass bleaching events, particularly within the last 15 years.

3) **Digital sampling approach** - We used a digital sampling approach for quantifying both corals and coral-associated fishes. Although quantifying coral cover from quadrats is common practice, quantifying reef fishes from photographs is a novel approach and provides several distinct advantages. It minimizes the so-called ‘diver effect’ on reef fishes, while maximizing the accuracy of fish counts. Commonly used underwater visual census (UVC) techniques, e.g. transects where the tape is laid first, have been shown to significantly underestimate the abundance of fishes through diver disturbance (38,39). Our photographs are taken within seconds of arriving at the quadrat location, thereby reducing diver-associated disturbance (2). Furthermore, in contrast to traditional UVC techniques, digital fish quantification is not constrained by time. For example, a single photograph may take up to 30 min to count and identify all fishes (e.g. max. 386 individuals per image). This approach, therefore, allows for all visible individuals to be counted, unlike traditional UVC techniques, which due to time constraints often have to estimate fishes in large schools. When needed, our approach also allows for the verification of fish species identity by multiple researchers. Finally, photographs are taken in high resolution, allowing us to identify even small recruits to species level (Supplementary Figure S2). Although quantifying fishes from photographs provides a single snapshot of fishes with some individuals potentially hidden from view behind coral branches, this effect is likely to be similar to live fish counts, where individuals may be missed if hiding deep within branches, diving for cover or swimming off while a diver is taking notes. Our approach is likely to be highly accurate for estimating small visually-apparent individuals.

*Methodological considerations*

We recognize the following potential limitations imposed by our sampling approach.

1) **Spatial scale, 1 m^2^ replicate quadrats** - In any spatial scale analysis, there will always be a trade-off in terms of ‘grain’ and ‘extent’, i.e. high-definition, detailed sampling over small scales, or less-detailed sampling over broader scales. We developed a sampling methodology at a grain of 1 m^2^ that allowed us to 1) directly examine fish-coral interactions at the scale of an individual fish’s daily activity, 2) simultaneously sample both fishes and corals in tight spatial associations and 3) 1 m^2^ quadrats could be repeatedly sampled across the timeframe of our study. Hence, this approach allowed us to assess widely held assumptions concerning the spatial match between fish and coral loss. In contrast, the majority of studies assessing the impacts of coral bleaching on reef fishes sample at a much larger grain and estimate patterns at a reef or reef-scape scale (see Supplementary Table S1).

We recorded all visually-apparent species in our 1 m^2^ quadrats. Hence, we did not exclude certain fish groups based on an *a priori* selection. Nevertheless, we noticed that larger species were relatively rare in our recordings. Surveying only a subset of the fish community, however, is common to all techniques, which typically do not record fishes in the recruit/juvenile category or cryptobenthic fishes, while others specifically target a particular species or family (e.g. Supplementary Table S1). Although our method may record only a few larger, roving fishes, we non-selectively recorded the entire visually apparent fish community present within each quadrat, incorporating both adult and juvenile fishes. Our approach was therefore designed to assess changes in fishes that closely associate with living coral, and that are expected to show pronounced declines in response to mass bleaching subsequent to live coral loss.

2) **Pre-disturbed system** - Lizard Island reefs constitute a pre-disturbed reef system. In addition to the 2016 and 2017 mass bleaching events (40), Lizard Island also sustained damage as a result of tropical cyclones (2014, 2015) (41,42) and an on-going series of crown-of-thorns starfish (CoTS) outbreaks (e.g. 33,43). Our ‘pre-bleaching’ surveys, therefore, were not taken from pristine, previously undisturbed reefs. This is, however, likely the case for the majority of coral reefs worldwide. It has been argued that there are no pristine reefs left in the world (44,45), as a result of previous and ongoing global pressures to reef ecosystems (e.g. mass bleaching of scleractinian corals, over-fishing, eutrophication, sedimentation, tropical storm damage, tourism, etc.), making such ‘pristine’ comparisons in the Anthropocene near impossible. To minimize the effects of cyclone damage, we only examined quadrats with a starting live coral cover of at least 20%.

3) **Single study location** - Our study surveyed reefs at a single location, Lizard Island. Unlike other studies, which typically assess changes in fishes and corals over large areas and often between different reefs or geographic locations (e.g. Supplementary Table S1), our study provides a ‘close-up’ perspective on key fish-coral interactions and mechanistic links, at a scale appropriate for examining fish behaviour. Our initial results, for example, revealed a strong spatial mismatch in fish and coral loss following the 2016 mass bleaching event, where fish losses were surprisingly not the highest in quadrats that experienced 1) the greatest loss of live coral or 2) a proliferation of cyanobacteria (2). Temporally-matched quadrats also revealed considerable post-bleaching increases in the abundance of coral-associated reef fishes, indicative of post-bleaching fish spatial movements (2). Although individual quadrat size was small (1 m^2^), quadrats were extensively replicated across the entire reef system of Lizard Island, which spans an area of over 10 km^2^. In this context, we therefore consider the surveyed area to be rather large, despite the restriction of a single location.

4) **Temporal scale** - In terms of scale, 24-mo is still in the realm of ‘short-term’ consequences (46), where the framework of dead corals has started to deteriorate, yet the underlying physical structure is still, to some degree, intact. In the long-term, consequences of mass bleaching are typically more pronounced, in comparison to short-term effects, due the loss of three-dimensional habitat structure (16,19). However, our focal fish community, coral-associated fishes, are thought to not only rely on the physical framework provided by corals, but also the live biological habitat it provides (33), and therefore typically show significant immediate and short-term declines in response to coral loss (e.g. 4). Moreover, the primary focus of the current study is to investigate the effects of bleaching on the recruits/juveniles of coral-associated fishes. Our sampling timeline encompasses two full recruitment cycles, and is similar in length to the average lifespan of many of our focal fishes. Given this context, a timeframe of 24-mo appears to be more than sufficient for the purpose of this study.

**Supplementary notes 2. Obligate live coral-dwelling fishes**

Live coral habitats provide critical ecosystem services to fishes that include food, settlement habitat and refuge from predators. Coral-dwelling is a particularly common trait in damselfishes (31,32), with an estimated 40% of damselfish species (GBR) associating with live corals as adults (34). Coral-associated fishes can be classified as 1) ‘facultative coral-dwellers’ - species that utilise live corals in an opportunistic manner or 2) ‘obligate live coral-dwellers’ - species with an obligate dependency on live coral (31). These classifications are generally based on the observed frequency of fishes associating with live coral hosts in nature (e.g. obligate coral-dweller: >95% (33); >80% (31) of all individuals observed per species) and disproportional declines in abundance following coral loss (32). Damselfishes consistently associate with corals that exhibit a branching morphology (31), i.e. primarily *Acropora* species, and to a lesser extent, species within *Echinopora*, *Pocillopora*, *Porites*, and *Seriatopora* (see Supplementary Table S2). The *degree* of live coral specialization, however, varies significantly among obligate coral-dwellers (33). *Dascyllus reticulatus*, for example, is a highly specialized species, documented to utilise just 8 species of corals, while *Pomacentrus moluccensis*, is less-specialized, and may use up to 30 different species of corals (33). Specialists, in comparison to generalists, are therefore more vulnerable to coral loss, especially if preferred coral species are susceptible to disturbance (13,31,34).

Across our 24-mo sampling period, total live coral cover decreased by 43.1%. The cover of *Acropora*, *Seriatopora* and *Stylophora* decreased to ≤ 0.1% cover per 1 m^2^, while *Pocillopora damicornis*, a preferred coral species by many obligate coral-dwelling damselfishes (e.g. *P. moluccensis*, *D. aruanus*, *D. reticulatus;* Pratchett et al. 2012), was rare, with a cover of < 1.0% across all sampling periods. Likewise, *Acropora valenciennesi*, the preferred coral species of *C. viridis* (33), was also rare and only recorded in a single quadrat. Remarkably, obligate coral-dwellers decreased a more moderate 46.8% (facultative: 32.4%) after 24-mo, despite the collapse of preferred corals. Given the strong associations between obligate live coral-dwellers and key coral genera/species, we expected a stronger response in these fishes, especially after two years, when the structural integrity of dead corals had begun to deteriorate.

Our results are in contrast to previous findings, which typically document stronger short-term declines in the abundance of obligate coral-dwelling fishes following localized coral loss. Commonly cited examples include (but see 15):

1. **GBR**: *P. moluccensis* (obligate) decreased by ~75% (mean) 12-mo after mass bleaching, in response to. a ~95% loss (mean) in pocilloporid cover (~70% mean loss in live coral cover) at affected sites (8).

2. **Tanzania**: *C. viridis* (obligate) decreased by 98% (mean) 6-mo post bleaching, in response to 100% *Acropora* loss (affected plots) (5).

3. **Japan**: *P. moluccensis* (obligate) decreased by 90% (mean) 12-mo after

mass bleaching in response to a 98% (mean) decrease in *Acropora* cover (4).

Furthermore, Pratchett and colleagues (33) documented a 30-70% loss in obligate coral-dwellers 11-mo after a COTS outbreak that decreased coral cover by <20% (consisting primarily of acroporids and pocilloporids). Losses of obligate coral-dwellers varied according to the degree of specialisation (i.e. number of coral species inhabited). Contrary to our results, obligate coral-dwellers showed little variation in microhabitat use between sampling years, i.e. continued to use branching coral genera, despite a lower availability of preferred corals post-CoTS outbreak. Pratchett et al. therefore suggested that the biological habitat provided by live corals, rather than the physical structure, may be the more critical component for obligate coral-dwellers. By contrast, obligate coral-dwellers in our study readily associated with a range of non-preferred microhabitats that included non-branching corals, dead corals, algal turf-covered areas and rubble.

**Supplementary notes 3. Live coral: key habitat for juvenile fishes**

Previous research has repeatedly emphasized the importance of live coral as a key habitat for recruits/juvenile reef fishes (e.g. 17,47-50), providing both shelter and critical settlement cues. It has been estimated, for example, that 65 % of reef fishes preferentially settle into (or near) living coral, even if they do not associate with live coral habitats as adults (17). Juvenile damselfishes, in particular, exhibit strong preferences for live coral (53% of species) and typically associate with either branching or plating growth forms (34). Live coral habitat acts as both a refuge for newly settled recruits and provides critical cues that encourage settlement (51). Therefore, it is both the loss of available live coral habitat to new recruits and the suppression of settlement cues associated with live coral that are expected to result in significant losses in fish abundance following coral loss (51). However, it should be noted that other factors such as the presence of conspecifics can also facilitate recruitment (52). To date, very few studies have investigated the responses of both newly settled recruits and juvenile reef fishes to mass bleaching in natural communities (Supplementary Table S1), although it has been suggested that microhabitat availability is a poor indicator of juvenile fish abundance at larger spatial scales (34). We show that the abundance of obligate coral-dwelling recruits/juveniles did not decrease in response to significant coral loss, despite the collapse of *Acropora* and other key coral genera. Recruits associated with a range of non-preferred microhabitats that included non-branching living corals and dead coral substrata.

**Supplementary notes 4. Other coral-dependent fishes**

Our initial analyses of ‘total’ fish abundances incorporated all fishes recorded within our replicate quadrats, which included over 150 fish species across 20 different fish families. Subsequently, we specifically focused our analyses on 27 species of damselfishes, which encompassed all species detected in our study with a reported facultative or obligate live coral association, based on 31. This emphasis on damselfishes was due to a number of reasons: 1) our novel sampling methodology is exceptionally well-suited for quantifying damselfish abundances; 2) damselfishes are important species that represent a key link between pelagic productivity and coral reefs and 3) damselfishes are a numerically dominant and conspicuous component of the fish assemblage on the GBR (e.g. they comprised over 60% of the total fish abundance in our data).

Other coral-dependent fishes with tight links to living corals include corallivorous butterflyfish and coral-dwelling cryptobenthic fishes, such as gobies. These fishes often show marked declines in abundance following extensive coral mortality, especially species with narrow habitat or dietary niches, e.g. 13,18,53. While recruitment dynamics of these fishes may have been affected in the timeframe of our study as a result of significant coral loss, we do not specifically assess changes in these fishes in the current study. Both butterflyfishes and cryptobenthic fishes represented a small component of our pre-bleaching fish assemblage (e.g. butterflyfishes made up just 0.6% of the pre-bleached fish abundance in our study), and have been extensively assessed in previous published studies. Commonly cited examples are summarized in Supplementary Table S6.

**Supplementary References**

1. Milicich, M.J., & Doherty, P.J. Larval supply of coral reef fish populations: magnitude and synchrony of replenishment to Lizard Island, Great Barrier Reef. *Mar. Ecol. Prog. Ser*. **110**, 121-121 (1994).

2. Wismer, S., Tebbett, S.B., Streit, R.P. & Bellwood, D.R. Spatial mismatch in fish and coral loss following 2016 mass coral bleaching. *Sci. Total Environ*. **650**, 1487-1498 (2019).

3. Wellington, G.M, & Victor, B.C. El Nino mass coral mortality: a test of resource limitation in a coral reef damselfish population. *Oecologia* 68, 15-19 (1985).

4. Shibuno, T., Hashimoto, K., Abe, O. & Takada, Y. Short-term changes in the structure of a fish community following coral bleaching at Ishigaki Island, Japan. *J Japan. Coral Reef Soc.*, *1999*, 51-58 (1999).

5. Lindahl, U.L.F., Öhman, M.C. & Schelten, C.K. The 1997/1998 mass mortality of

corals: effects on fish communities on a Tanzanian coral reef." *Mar. Poll. Bull.* ***42***, 127-131 (2001).

6. Kokita, T., & Nakazono, A. Rapid response of an obligately corallivorous filefish

*Oxymonacanthus longirostris* (Monacanthidae) to a mass coral bleaching event. *Coral Reefs*, *20*(2), 155-158 (2001).

7*.* Adjeroud, M., Augustin, D., Galzin, R., & Salvat, B. Natural disturbances and interannual variability of coral reef communities on the outer slope of Tiahura (Moorea, French Polynesia): 1991 to 1997. *Mar. Ecol. Prog. Ser.* **237**, 121-131 (2002).

8. Booth, D.J. & Beretta, G.A. Changes in a fish assemblage after a coral bleaching event. *Mar. Ecol. Prog. Ser*. **245**, 205-212 (2002).

9. McClanahan, T, Maina, J & a Pet-Soede. L. Effects of the 1998 coral morality event on Kenyan coral reefs and fisheries. *Ambio* **31**, 543-551 (2002).

10. Riegl, B. Effects of the 1996 and 1998 positive sea-surface temperature anomalies on corals, coral diseases and fish in the Arabian Gulf (Dubai, UAE). *Mar. Biol.* **140**, 29-40 (2002).

11. Spalding, M.D., & Jarvis, G.E. The impact of the 1998 coral mortality on reef fish communities in the Seychelles. *Mar. Poll. Bull.* ***44***, 309-321 (2002).

12. Sheppard, C.R., Spalding, M., Bradshaw, C., & Wilson, S. Erosion vs. recovery of

coral reefs after 1998 El Niño: Chagos reefs, Indian Ocean. *Ambio* **31**, 40-49 (2002).

13. Munday, P.L. Habitat loss, resource specialization, and extinction on coral reefs.

*Global Chang. Biol*. **10**, 1642-1647 (2004).

14. Pratchett, M.S., Wilson, S.K., Berumen, M.L. & McCormick, M.I. Sublethal effects of coral bleaching on an obligate coral feeding butterflyfish. *Coral Reefs* **23**, 352-356 (2004).

15. Sano, M. Short-term effects of a mass coral bleaching event on a reef fish assemblage at Iriomote Island, Japan. *Fish. Sci.* ***70***, 41-46 (2004).

16. Garpe, K. C., Yahya, S. A., Lindahl, U. & Öhman, M. C. Long-term effects of the 1998 coral bleaching event on reef fish assemblages. *Mar. Ecol. Prog. Ser.***315**, 237-247 (2006).

17. Jones, G.P., McCormick, M.I., Srinivasan, M. & Eagle, J.V. Coral decline threatens fish biodiversity in marine reserves. *Proc. Nat. Acad. Sci.* **101**, 8251-8253 (2004).

18. Pratchett, M.S., Wilson, S.K. & Baird, A.H. Declines in the abundance of *Chaetodon* butterflyfishes following extensive coral depletion. *J. Fish Biol.* **69**, 1269-1280 (2006).

19. Graham et al. N.A.J. Lag effects in the impacts of mass coral bleaching on coral reef fish, fisheries, and ecosystems. *Conserv. Biol.* **21**, 1291-1300 (2007).

20. Wilson, S.K. et al. Exploitation and habitat degradation as agents of change within coral reef fish communities. *Glob. Chang. Biol*. **14**, 2796-2809 (2008).

21. Graham, N.A.J., Wilson, S.K., Pratchett, M.S., Polunin, N.V.C., & Spalding, M.D. Coral mortality versus structural collapse as drivers of corallivorous butterflyfish decline. *Biodivers. Consev*, ***18***, 3325-3336 (2009).

22. Gilmour, J.P., Smith, L.D., Heyward, A.J., Baird, A.H., & Pratchett, M.S. Recovery of an isolated coral reef system following severe disturbance. *Science*, **340**, 69-71 (2013).

23. Brooker, R.M., Munday, P.L., Brandl, S.J., & Jones, G.P. Local extinction of a coral reef fish explained by inflexible prey choice. *Coral Reefs* **33**, 891-896 (2014).

24. Glynn, P.W., Enochs, I.C., Afflerbach, J.A., Brandtneris, V.W., & Serafy, J.E. Eastern Pacific reef fish responses to coral recovery following El Niño disturbances. *Mar. Ecol. Prog. Ser.* **495**, 233-247 (2014).

25. Mangubhai, S., Strauch, A.M., Obura, D.O., Stone, G., & Rotjan, R.D. Short-term changes of fish assemblages observed in the near-pristine reefs of the Phoenix Islands. *Rev. Fish Biol. Fisheries* **24**, 505-518 (2014).

26. Richardson, L.E., Graham, N.A., Pratchett, M.S., Eurich, J.G., & Hoey, A.S. Mass coral bleaching causes biotic homogenization of reef fish assemblages. *Glob. Chang. Biol.*  **24**, 3117-3129 (2018).

27. Stuart-Smith, R.D. et al. Ecosystem restructuring along the Great Barrier Reef following mass coral bleaching. *Nature* **560**, 92 (2018).

28. Keith, S.A. et al., Synchronous behavioural shifts in reef fishes linked to mass coral bleaching. *Nat. Clim. Change* ***8***, 986-991 (2018).

29. Wilson, S. K., Robinson, J. P., Chong-Seng, K., Robinson, J., & Graham, N. A. Boom and bust of keystone structure on coral reefs. *Coral Reefs*, 1-11 (2019).

30. McClure, E.C. et al. Cross-Shelf Differences in the Response of Herbivorous Fish Assemblages to Severe Environmental Disturbances. *Diversity* **11**, 23 (2019).

31. Pratchett, M.S., Hoey, A.S., Wilson, S.K., Hobbs, J.P & Allen, G. Habitat-use and specialisation among coral reef damselfishes. In: Biology of Damselfishes (B. Frédérich & E. Parmentier, Eds). CRC Press, Boca Raton: pp. 84-121 (2016).

32. Coker, D.J., Wilson, S.K. & Pratchett, M.S. Importance of live coral habitat for reef fishes. *Rev. Fish Biol. Fisher.* **24**, 89-126 (2013).

33. Pratchett, M.S., Coker, D.J., Jones, G.P. & Munday, P.L. Specialization in habitat use by coral reef damselfishes and their susceptibility to habitat loss. *Ecol. Evol.***2**, 2168-2180 (2012).

34. Wilson, S.K. et al. Habitat utilization by coral reef fish: implications for specialists vs. generalists in a changing environment. *J. Anim. Ecol*. ***77***, 220-228 (2008).

35. Berumen, M.L. & Pratchett, M.S. Recovery without resilience: persistent disturbance and long-term shifts in the structure of fish and coral communities at Tiahura Reef, Moorea. *Coral Reefs* ***25***, 647-653 (2006).

36. Bellwood, D.R., Hoey, A.S., Ackerman, J.L. & Depczynski. M. Coral bleaching, reef fish community phase shifts and the resilience of coral reefs. *Global Change Biol.* ***12***, 1587-1594 (2006).

37. Bellwood, D.R. et al., Coral recovery may not herald the return of fishes on damaged coral reefs. *Oecologia* ***170***, 567-573 (2012).

38. Dickens, L.C., Goatley, C.H., Tanner, J.K & Bellwood, D.R. Quantifying relative diver effects in underwater visual censuses. *Plos One* **6**, e18965 (2011).

39. Emslie, M.J., Cheal, A.J., MacNeil, M.A., Miller, I.R. & Sweatman, H.P.A. Reef fish communities are spooked by scuba surveys and may take hours to recover. Peer J ***6***, e4886 (2018).

40. Hughes, T.P. et al. Ecological memory modifies the cumulative impact of recurrent climate extremes. *Nat. Clim. Chang.* **9**, 40 (2019).

41. Ceccarelli, D.M., Emslie, M.J. & Richards, Z.T. Post-disturbance stability of fish assemblages measured at coarse taxonomic resolution masks change at finer scales. *PLoS One* **11.6**, e0156232 (2016).

42. Madin, J.S. et al. Cumulative effects of cyclones and bleaching on coral cover and species richness at Lizard Island. *Mar. Ecol. Prog. Ser.* **604**, 263-268 (2018).

43. Pratchett, M.S. Changes in coral assemblages during an outbreak of *Acanthaster planci* at Lizard Island, northern Great Barrier Reef (1995–1999). *Coral Reefs* ***29,*** 717-725 (2010).

44. Hughes, T.P. et al. Climate change, human impacts, and the resilience of coral reefs. *Science***301**, 929-933 (2003).

45. Hughes, T.P. et al. Coral reefs in the Anthropocene. *Nature***546**, 82 (2017).

46. Pratchett, M.S. et al. Coral bleaching and consequences for motile reef organisms: past, present and uncertain future effects. In: *Coral Bleaching*. Springer, Berlin: pp. 139-158 (2009).

47. Boström-Einarsson, L. et al., Loss of live coral compromises predator-avoidance behaviour in coral reef damselfish. *Sci. Rep*. ***8***, 7795 (2018).

48. Dixon, D.L., Abrego, D. & Hay, M.E. Chemically mediated behavior of recruiting corals and fishes: a tipping point that may limit reef recovery. *Science***345**, 892-897 (2014).

49. Feary, D.A. et al., Habitat choice, recruitment and the response of coral reef fishes to coral degradation. *Oecologia* ***153***, 727-737 (2007).

50. Feary, D.A., McCormick, M.I., & Jones, G.P. Growth of reef fishes in response to live coral cover. *J.* *Exp. Mar. Biol. Ecol*. **373**, 45-49 (2009).

51. Munday, P.L. et al., Climate change and the future for coral reef fishes. *Fish Fish.* ***9***, 261-285 (2008).

52. Sweatman, H.P.A. The influence of adults of some coral reef fishes on larval recruitment. *Ecol. Monogr.* **55**, 469-485 (1985).

53. Pratchett, M.S. Effects of climate-induced coral bleaching on coral-reef fishes -ecological and economic consequences. *Oceanogr. Mar. Biol. An. Rev* **46,** 257-302 (2008).
