# Supplementary material for: Young fishes persist despite coral loss on the Great Barrier Reef
Source: Commun Biol. 2019 Dec 6;2:456. doi: 10.1038/s42003-019-0703-0 (PMC6898333; doi:10.1038/s42003-019-0703-0)
Supplement: Supplementary file 2 — Description of Additional Supplementary Files [file 42003_2019_703_MOESM2_ESM.pdf]

The supplementary data 1 file contains the data underlying the figures in the main manuscript and includes total fish abundance, the abundance of all obligate/facultative coral associated damselfishes, the abundance of obligate/facultative coral associated damselfish recruits, cover of *Acropora* corals and total coral cover. Data is across four sampling trips (A, B, C, D) and all data is at the level of individual quadrats (1 m^2).
