# Supplementary material for: Young fishes persist despite coral loss on the Great Barrier Reef
Source: Commun Biol. 2019 Dec 6;2:456. doi: 10.1038/s42003-019-0703-0 (PMC6898333; doi:10.1038/s42003-019-0703-0)
Supplement: Supplementary file 4 — Reporting Summary [file 42003_2019_703_MOESM4_ESM.pdf]

nature research

Corresponding author(s): David R. Bellwood

Last updated by author(s): Oct 28, 2019

Reporting Summary

Nature Research wishes to improve the reproducibility of the work that we publish. This form provides structure for consistency and transparency in reporting. For further information on Nature Research policies, see Authors & Referees and the Editorial Policy Checklist.

Statistics

For all statistical analyses, confirm that the following items are present in the figure legend, table legend, main text, or Methods section.

n/a	Confirmed	
☐	☒	The exact sample size (*n*) for each experimental group/condition, given as a discrete number and unit of measurement
☐	☒	A statement on whether measurements were taken from distinct samples or whether the same sample was measured repeatedly
☐	☒	The statistical test(s) used AND whether they are one- or two-sided<br>*Only common tests should be described solely by name; describe more complex techniques in the Methods section.*
☐	☒	A description of all covariates tested
☐	☒	A description of any assumptions or corrections, such as tests of normality and adjustment for multiple comparisons
☐	☒	A full description of the statistical parameters including central tendency (e.g. means) or other basic estimates (e.g. regression coefficient) AND variation (e.g. standard deviation) or associated estimates of uncertainty (e.g. confidence intervals)
☐	☒	For null hypothesis testing, the test statistic (e.g. *F*, *t*, *r*) with confidence intervals, effect sizes, degrees of freedom and *P* value noted<br>*Give P values as exact values whenever suitable.*
☒	☐	For Bayesian analysis, information on the choice of priors and Markov chain Monte Carlo settings
☒	☐	For hierarchical and complex designs, identification of the appropriate level for tests and full reporting of outcomes
☒	☐	Estimates of effect sizes (e.g. Cohen's *d*, Pearson's *r*), indicating how they were calculated

Our web collection on statistics for biologists contains articles on many of the points above.

Software and code

Policy information about availability of computer code

Data collection	We quantified changes in coral cover using a software called photoQuad, which generates randomly stratified points over each 'planar perspective' photoquadrat.
Data analysis	All statistical modelling was performed in the software R, using the lme4 and glmmTMB packages.

For manuscripts utilizing custom algorithms or software that are central to the research but not yet described in published literature, software must be made available to editors/reviewers. We strongly encourage code deposition in a community repository (e.g. GitHub). See the Nature Research guidelines for submitting code & software for further information.

Data

Policy information about availability of data

All manuscripts must include a data availability statement. This statement should provide the following information, where applicable:

- Accession codes, unique identifiers, or web links for publicly available datasets
- A list of figures that have associated raw data
- A description of any restrictions on data availability

The data that support the findings of this study are available from the corresponding author upon request.

Field-specific reporting

Please select the one below that is the best fit for your research. If you are not sure, read the appropriate sections before making your selection.

☐ Life sciences ☐ Behavioural & social sciences ☒ Ecological, evolutionary & environmental sciences

Ecological, evolutionary & environmental sciences study design

All studies must disclose on these points even when the disclosure is negative.

Study description	To quantify changes in the cover of live coral and coral-associated fishes, in response to mass coral bleaching, we compared data from a total of 132 photoquadrats, repeatedly sampled across 24 months. Specifically, in each field trip, we sampled 19 transect sites across the reefal system of Lizard Island. Transects varied in length depending on reef length, i.e. range 50-210 m. Within each transect, photographs of a 1 m2 quadrat were taken at approx. 5 m intervals, i.e. range per transect, 12-38 quadrats depending on transect length. Each specific quadrat location was photographed 4 times: 1) Jan. 2016 – before mass bleaching; 2) April 2016 – peak bleaching, 3) Oct. 2016 – 6-mo post bleaching, 4) Jan. 2018 – 24-mo since first sampling trip. Changes in coral cover and associated fish assemblage were then compared between sampling trips, in relation to the 2016-2017 mass bleaching events.
Research sample	Our research samples were replicate 1 m2 quadrats. In total, we analysed 132 individual 1 m2 quadrats and each specific quadrat location was sampled four times across 24-months. Within each quadrat, we 1) quantified the percent cover of benthic categories (e.g. live coral cover) and 2) identified and recorded all visible fishes, which consisted primarily of small, coral-associated fishes. Although quantifying coral cover from quadrats is common practice, quantifying reef fishes from photographs is a novel approach and provides several distinct advantages, including 1) reduced diver disturbance to fish communities and 2) no time constraints when quantifying fishes.
Sampling strategy	Quadrats were spread across the entire lagoon and fringing reefs of the sampling location, Lizard Island. Quadrats were selected if they had greater than 20% coral cover (based on visual estimates) as the focus of the paper was on fish responses to coral loss. The number of quadrats was not set a priori but depended on the initial results. Of the 451 quadrats examined 132 had the requisite starting coral cover and were subsequently examined throughout the 24 month period.
Data collection	Our data collection procedure involved both a field and lab component. During the field component, we collected a series of photographs of a 1 m2 quadrat at 451 quadrat locations (132 of 451 were analysed). Specifically, at each quadrat location, we collected three images: 1) an undisturbed horizontal perspective photograph of the reef and coral-associated reef fishes (at a distance of 2 m), taken within seconds of reaching the site and prior to the placement of the quadrat, 2) a second horizontal perspective photograph with the 1 m2 quadrat in place, using the identical camera placement as in the first image, and 3) a planar perspective photograph (i.e. bird's-eye view) of the 1 m2 quadrat in place over the substratum. Images were subsequently examined back in the laboratory using standard computer-based image analysis. Coral cover was quantified using the software photoQuad, which generates 40 random stratified data points over the image, from which we calculated percent cover. All corals were quantified to species level when possible, categorized by state, i.e. living, bleached etc. To quantify reef fishes, photoquadrats were processed in Adobe Illustrator, by drawing an outline of the quadrat on the first photograph of the series (i.e. undisturbed), using the second photograph in the series as a reference. All visible reef fishes within the delineated 1 m2 (and 1.5 m above the quadrat) were recorded to species level and categorized as either adult or recruit. All field photographs were taken by R.S. or S.T. All images were processed by one person for consistency (S.T.).
Timing and spatial scale	To quantify changes in coral cover and the associated fish assemblage, as a result of mass bleaching, we carefully timed our sampling trips across a 24-mo timeline. The first sampling trip was conducted in Jan. 2016, immediately before the 2016 mass bleaching event. Data from this sampling trip would serve as a baseline. To quantify the extent of bleaching, we re-sampled the same sites in April 2016, at the peak of the mass bleaching event. All sites were once again sampled in Oct. 2016, 6-mo post bleaching and Jan. 2018, 24-mo post bleaching, in order to quantify both short and long-term changes in both coral and fish communities, in response to mass bleaching. The duration of each sampling trip was approx. 2 weeks. All samples were collected from reefs surrounding Lizard Island, which was near the GBR epicenter of the 2016 global mass bleaching event.
Data exclusions	Per sampling period, a total of 132 (out of 451) 1 m2 photoquadrats were analysed. Since we were explicitly examining the response of coral-associated reef fishes (i.e. fishes with a facultative or obligate so-called dependency on live coral) to mass bleaching, we only analysed quadrats with a minimum live coral cover of 20% (based on visual estimates) in the first sampling period. For analyses on benthic cover, points which were categorized as 'unidentifiable', were excluded from analyses. This number, however, was very low, i.e. 16 of 21,120 benthic points examined were excluded.
Reproducibility	Our study quantifies changes in both live coral cover and coral-associated fishes across the 2016 global mass bleaching event, a natural phenomenon that cannot be replicated. However, our results are directly comparable to other studies of mass bleaching events.<br><br>One potential issue in our sampling design is is quadrat placement, i.e. sampling the same exact site across all trips. The effect of random sampling, as well as, variation in quadrat placement, was assessed by examining 40 random points from the first sampling period versus the exact same 40 points from the third sampling period (n = 15 randomly selected quadrats). The results showed just a 1.4% difference, and hence, our method appears to provide a good indication of benthic changes among temporal samples with excellent reproducibility.
Randomization	The starting locations of each of the 19 surveyed transect sites were chosen haphazardly in the first sampling trip, i.e. we did not focus our efforts in areas of the reef which were more prone to bleaching or that exhibited higher bleaching rates in past. Transects were spread across the reefal system of Lizard Island.
Blinding	Since we were documenting a natural phenomenon, blinding is not possible.

Did the study involve field work? ☒ Yes ☐ No

Field work, collection and transport

Field conditions	The reefs of Lizard Island were assessed in 4 separate sampling trips: 1) Jan. 2016 – before mass bleaching; 2) April 2016 – peak bleaching, 3) Oct. 2016 – 6-mo post bleaching, and 4) Jan. 2018 – 24-mo since first sampling trip. During this timeframe, the Great Barrier Reef experienced unprecedented back-to-back mass bleaching of scleractinian corals (i.e. Feb. - April 2016; Jan. – March 2017), as a result of prolonged elevated sea-surface temperatures during marine heatwaves. Sea surface temperatures for Feb., March and April 2016 were the hottest on record, with mean sea surface temperatures recorded at 29.1, 29.1 and 27.8C, respectively. Sea surface temperatures for Jan, Feb. and March 2017 were slightly cooler that the 2016 marine heatwave, but still significantly warmer than long-term monthly averages, at 28.8, 28.8 and 28.7C, respectively.
Location	This study was conducted at 19 reef transect sites Lizard Island, in the northern region of Great Barrier Reef, Queensland, Australia (14°40'S, 145°28'E). In total, we surveyed 19 individual transect sites. Transects were always placed along the reef crest habitat, at 0 – 4 m below chart-datum. Within each transect, we took photographs of a 1 m2 quadrat at approximately 5m intervals, ranging from 12 to 38 quadrats per individual transect. Each specific transect location was photographed four times in our 24-mo study timeframe.
Access and import/export	No material was removed and all access was covered by Great Barrier Reef Marine Park Authority permits.
Disturbance	Our study was observational in nature and and therefore, disturbance to the reef was negligable. Our methodology involved swimming a transect along the reef crest and placing and photographing a 1 m2 quadrat repeatedly along the transect/reef crest. Quadrats were always placed carefully over the reef substratum, to not damage live coral or other benthic organisms. Disturbance to the fish community, as a result of SCUBA diver presence, was minimal. Divers were only in a single quadrat locations for a few minutes, and fish activity returned to normal quickly thereafter.

Reporting for specific materials, systems and methods

We require information from authors about some types of materials, experimental systems and methods used in many studies. Here, indicate whether each material, system or method listed is relevant to your study. If you are not sure if a list item applies to your research, read the appropriate section before selecting a response.

Materials & experimental systems

n/a	Involved in the study
☒	☐ Antibodies
☒	☐ Eukaryotic cell lines
☒	☐ Palaeontology
☐	☒ Animals and other organisms
☒	☐ Human research participants
☒	☐ Clinical data

Methods

n/a	Involved in the study
☒	☐ ChIP-seq
☒	☐ Flow cytometry
☒	☐ MRI-based neuroimaging

Animals and other organisms

Policy information about studies involving animals; ARRIVE guidelines recommended for reporting animal research

Laboratory animals	Our study was strictly field-based and did not utilize laboratory animals in experiments.
Wild animals	Our study was observation-based and did not require handling, manipulation, transport or killing of wild animals. We quantified changes in 1) the abundance of coral-associated fishes and 2) coral cover strictly from photographs taken in the field across multiple sampling periods.
Field-collected samples	Our field-collected samples were restricted to photographs taken of individual quadrat locations at Lizard Island, which were copied and transported back to the laboratory on external hard drives.
Ethics oversight	No ethics permission was required as this was a minimum disturbance observational study of invertebrates and fish.

Note that full information on the approval of the study protocol must also be provided in the manuscript.
